# Characterization of novel anoikis-related genes as prognostic biomarkers and key determinants of the immune microenvironment in esophageal cancer

**DOI:** 10.3389/fimmu.2025.1599171

**Published:** 2025-07-11

**Authors:** Yani Su, Ming Zhang, Peng Xu, Pengfei Wen, Ke Xu, Jiale Xie, Xianjie Wan, Lin Liu, Zhi Yang, Mingyi Yang

**Affiliations:** ^1^ Department of Radiotherapy, Tangdu Hospital, Fourth Military Medical University, Xi’an, Shaanxi, China; ^2^ Department of General Practice, Honghui Hospital, Xi’an Jiaotong University, Xi’an, Shaanxi, China; ^3^ Department of Joint Surgery, HongHui Hospital, Xi’an Jiaotong University, Xi’an, Shaanxi, China

**Keywords:** esophageal cancer, anoikis, prognosis, tumor microenvironment, immune

## Abstract

**Objective:**

Esophageal cancer (EC) ranks among the most prevalent malignancies globally and represents a significant and growing public health burden. This study aimed to construct a prognostic model leveraging anoikis-related genes (ARGs) to predict patient survival and elucidate the immunological microenvironment in EC. The findings are anticipated to enhance prognostic accuracy and inform therapeutic strategies, ultimately improving patient outcomes and treatment efficacy.

**Methods:**

A comprehensive analysis was conducted using 11 control samples and 159 EC samples obtained from The Cancer Genome Atlas (TCGA) database, alongside associated clinical features. A total of 794 ARGs were curated from GeneCards database. Functional enrichment analyses of EC-related differentially expressed ARGs were performed using Gene Ontology (GO) and the Kyoto Encyclopedia of Genes and Genomes (KEGG). Prognostic differential ARGs associated with EC were identified through univariate Cox regression analysis, while LASSO regression was employed to minimize overfitting and construct a robust risk prognostic model. The EC cohort was stratified into training and testing groups for model development and verification. Model performance was evaluated through risk curves, survival curves, time-dependent receiver operating characteristic (ROC) curves, ROC curves for the riskscore and clinical features, and independent prognostic analysis. A nomogram with high predictive accuracy was also developed to estimate the prognosis of EC patients. To assess the impact of the risk prognosis model on the immune microenvironment of EC, analyses included tumor microenvironment analysis, single-sample gene set enrichment analysis (ssGSEA), immune cell infiltration correlation analysis, and differential analysis of immune checkpoint expression. Drug sensitivity profiling was conducted to identify potential therapeutic agents for EC. Finally, the expression of selected ARGs was validated at the mRNA level in EC cell lines using real-time quantitative PCR (RT-qPCR).

**Results:**

The ARG-based risk prognostic model was constructed incorporating four high-risk ARGs (CDK1, IL17A, FOXC2, and OLFM3) and two low-risk ARGs (PIP5K1C and MAPK1). This model demonstrated strong predictive accuracy for the survival outcomes of EC patients. Immune correlation analyses revealed that the high-risk group exhibited significantly lower immunological scores compared to the low-risk group. Notably, immune cells such as macrophages and mast cells were markedly downregulated in the high-risk group. Additionally, key immunological functions, including APC co-inhibition, parainflammation, Type I IFN Response, and Type II IFN Response, were significantly suppressed in the high-risk group. Eight immune checkpoint-related genes (TNFRSF25, TNFRSF14, CD70, TNFSF15, TMIGD2, CD160, TNFSF18, and HHLA2) displayed distinct expression differences between high- and low-risk groups. The nomogram developed from this model demonstrated high efficacy in predicting EC patient prognosis. Furthermore, six potential therapeutic agents for EC were identified: BIRB.0796, Camptothecin, CHIR.99021, Methotrexate, PF.4708671, and Vorinostat. Finally, the mRNA expression levels of ARGs were validated using RT-qPCR in EC cell lines. Compared to normal esophageal epithelial cells (NE-2), CDK1 and MAPK1 were significantly upregulated in two EC cell lines (KYSE-30 and KYSE-180).

**Conclusion:**

This study provides valuable insights into the prognostic outcomes and immune microenvironment of EC through the analysis of ARGs. Furthermore, several potential therapeutic agents for EC were identified, offering promising avenues for treatment. These findings hold significant potential for enhancing the survival outcomes of EC patients and provide meaningful guidance for clinical decision-making in managing this malignancy.

## 1 Introduction

Esophageal cancer (EC) ranks among the most prevalent and lethal malignancies globally, posing an increasingly significant disease burden worldwide (1). Despite advancements in diagnostic and therapeutic approaches, the prognosis for EC patients remains poor, largely due to the absence of robust and reliable diagnostic biomarkers. Although surgical resection remains a cornerstone of treatment and can extend patient survival, high rates of recurrence and metastasis continue to challenge clinical management, significantly limiting long-term outcomes (2). Understanding the molecular and cellular mechanisms underlying EC progression is therefore critical to addressing these challenges. Insights into these mechanisms could facilitate the identification of novel diagnostic indicators and therapeutic targets, ultimately improving early detection, treatment efficacy, and patient survival rates (1, 3). This underscores the urgent need for continued research into the pathogenesis and progression pathways of EC to inform innovative clinical strategies and reduce the global burden of this malignancy.

Anoikis is a specialized form of programmed cell death triggered by the disruption of cell-cell or cell-extracellular matrix (ECM) attachments. This process is essential for maintaining tissue homeostasis, as it eliminates misplaced or detached cells, thereby preventing inappropriate cellular growth and localization (4, 5). Anoikis plays a pivotal role in various physiological and pathological processes, including development, carcinogenesis, and the maintenance of tissue equilibrium (6). In most cancers, anchorage-dependent growth is a hallmark, and the absence of ECM attachment typically induces anoikis, which serves as a critical barrier to tumor metastasis. Consequently, the activation of anoikis is a vital mechanism in counteracting tumor initiation and progression (5). However, for cancer cells to metastasize successfully, they must evade anoikis. Evidence from prior research highlights the significance of this evasion in cancer biology. For example, inactivation of IL1RAP induces anoikis and prevents the metastatic spread of Ewing sarcoma cells (5). Similarly, Tubeimoside V sensitizes triple-negative breast cancer MDA-MB-231 cells to anoikis by modulating caveolin-1-related signaling pathways (4), and disulfiram activates calpain-mediated anoikis, inhibiting lung colonization in triple-negative breast cancer (5, 7). Unfortunately, not all cancer cells are susceptible to anoikis, as some acquire resistance, a phenomenon crucial to the progression of certain malignancies (8, 9). For instance, HCRP-1 regulates anoikis resistance through the EGFR-AKT-BIM axis and serves as a prognostic marker in colon cancer (10). The PLAG1-GDH1 axis promotes anoikis resistance and metastasis via CAMKK2-AMPK signaling in LKB1-deficient lung cancer (9). Moreover, nuclear MYH9-induced CTNNB1 transcription, which can be targeted by staurosporine, enhances anoikis resistance and metastasis in gastric cancer cells (11). Despite these insights into the role of anoikis in various cancers, its function and mechanisms in EC remain unexplored. This gap underscores the need for focused research to elucidate the involvement of anoikis in EC progression and its potential as a therapeutic target.

The development of robust risk prognostic models holds significant promise for predicting tumor outcomes and improving personalized treatment strategies. In recent years, novel prognostic models have been proposed to enhance the accuracy of predicting patient prognosis across various cancers. For instance, a ferroptosis-related lncRNA signature has been shown to correlate with esophageal squamous cell carcinoma (ESCC) prognosis, tumor microenvironment dynamics, and therapeutic responsiveness (12). Similarly, an autophagy-related gene signature has been identified and validated for its prognostic value in ESCC patients, providing insights into tumor progression and patient outcomes (13). Moreover, transcriptional and genomic alterations in cuproptosis-related genes have been linked to EC malignancy and immune infiltration, highlighting their potential as biomarkers and therapeutic targets (14). Building upon this foundation, the current study introduces a novel risk prognostic model based on ARGs, which offers a new perspective on EC prognosis. This model not only underscores the critical role of ARGs in predicting patient outcomes but also sheds light on their influence within the immune microenvironment. By integrating ARGs into the prognostic framework, this study provides a deeper understanding of their involvement in tumor progression, immune regulation, and potential therapeutic interventions. Such advancements pave the way for more precise risk stratification and personalized treatment strategies for EC patients, ultimately aiming to improve clinical outcomes and quality of life.

## 2 Materials and methods

### 2.1 Data acquisition and collation

The transcriptomic and clinical data utilized in this study were sourced from The Cancer Genome Atlas (TCGA) database (https://portal.gdc.cancer.gov/), comprising a total of 170 samples, including 11 normal controls and 159 EC samples. The clinical parameters for EC patients included survival time (futime), survival status (fustat), gender, pathological stage, and TNM classification (T: tumor size/invasion, N: lymph node involvement, M: distant metastasis). Additionally, a comprehensive set of 794 ARGs was curated from the GeneCards database (https://www.genecards.org/). From the GeneCards database, we retrieved ARGs associated with “Anoikis” categorized as “Protein Coding”.

### 2.2 EC-related differentially expressed ARGs

To identify ARGs associated with EC, a set of 794 ARGs was intersected with the genes expressed in the EC transcriptomic dataset. Differential expression analysis was conducted on 159 EC samples and 11 normal control samples using the limma package in R. The analysis identified differentially expressed genes (DEGs) in EC based on the screening thresholds of P<0.05 and |logFC|≥0.5 (15). Subsequently, the intersection of EC-related ARGs with EC-specific DEGs yielded a subset of differentially expressed ARGs associated with EC, providing a focused set of candidates for further investigation.

### 2.3 Enrichment analysis

The Database for Annotation, Visualization, and Integrated Discovery (DAVID, http://david.abcc.ncifcrf.gov/) was employed to conduct Gene Ontology (GO) and Kyoto Encyclopedia of Genes and Genomes (KEGG) enrichment analyses for the differentially expressed ARGs associated with EC. A significance threshold of P<0.05 was applied for screening (16). GO enrichment analysis encompassed three primary categories: Biological Processes (BP), Cellular Components (CC), and Molecular Functions (MF), providing a comprehensive framework for understanding the functional roles of these genes in EC pathogenesis.

### 2.4 Construction of risk prognostic model

Univariate Cox regression analysis was performed using the survival package in R to identify the differentially expressed ARGs significantly associated with EC prognosis. To mitigate the risk of overfitting and determine the optimal number of differentially expressed ARGs for model construction, LASSO regression analysis was subsequently conducted using the glmnet package in R (17). The EC samples were randomly partitioned into training and testing cohorts, and a prognostic risk model was developed. The riskscore for each patient was then calculated as follows:


$$\text{Riskscore}=\sum_{i=1}^{n}\left(mrnaexp_{i}\times coef_{i}\right)$$

The n represents the number of differentially expressed ARGs associated with EC prognosis, and i denote the i-th ARG. The riskscore for each sample was calculated by multiplying the expression level of each EC prognosis-related differentially expressed ARGs by its corresponding regression coefficient, and then summing the results (18). Based on the median riskscore, EC patients were stratified into high-risk and low-risk groups for further analysis.

### 2.5 Validation of the risk prognostic model

To assess the differential survival outcomes between high- and low-risk groups, a risk curve and survival status plot were generated using R, enabling visualization of survival status variations among EC patients (19). The pheatmap package in R was utilized to create a heatmap, which allowed for the examination of the expression patterns of differentially expressed ARGs in both high- and low-risk groups. To evaluate the prognostic impact of the risk model on EC patient survival, survival analysis was conducted using the survival and survminer packages in R. Time-dependent receiver operating characteristic (ROC) curves were generated to assess the model’s predictive accuracy over time, as well as ROC curves for the riskscore and clinical features, using the survival, survminer, and timeROC packages. Furthermore, to determine whether the risk score and clinical features could serve as independent prognostic factors, univariate and multivariate Cox regression analyses were performed using the survival package in R. These analyses provided a robust evaluation of the model’s potential utility in clinical prognostication.

### 2.6 Risk differential analysis

Differential analysis between the high-risk and low-risk groups within both the training and testing cohorts was conducted using the reshape2 and ggpubr packages in R. This analysis aimed to evaluate whether the ARGs incorporated in the model exhibited distinct expression patterns between the two risk groups.

### 2.7 Clinical features analysis

A nomogram is a valuable tool for predicting cancer prognosis, and in this study, we developed a nomogram based on the training cohort. The RMS package in R was utilized to construct the nomogram, which was designed to predict the 1-, 3-, and 5-year survival outcomes of EC patients. Additionally, a calibration curve was plotted to assess the agreement between the predicted and observed survival probabilities. To evaluate the applicability of the risk prognostic model across various clinical subgroups of EC patients, we validated its performance according to different clinical features. The clinical features were categorized as follows: gender (male *vs*. female), tumor stage (I-II *vs*. III-IV), T stage (T1–2 *vs*. T3-4), N stage (N0 *vs*. N1-3), and M stage (M0 *vs*. M1). Model validation was performed using the survival and survminer packages in R to test whether the prognostic model was applicable and effective within each clinical subgroup. This validation allowed us to assess the robustness and generalizability of the model in predicting survival outcomes across diverse patient profiles.

### 2.8 Tumor microenvironment analysis

Tumor microenvironment analysis was conducted on the EC transcriptome data using the limma and estimation in R. This analysis yielded stromal scores, immune scores, and the ESTIMATE score for each EC patient. The stromal, immune, and ESTIMATE scores for both the high-risk and low-risk groups within the training cohort were subsequently examined using the limma and ggpubr R packages, allowing for a comparative evaluation of tumor microenvironment components across the risk groups.

### 2.9 Single sample gene set enrichment analysis (ssGSEA)

The ssGSEA of the EC transcriptome data was conducted using the GSVA, limma, and GSEABase R packages to calculate enrichment scores for immune cell types and immune functions. Subsequently, the limma, reshape2, and ggpubr packages in R were utilized to assess and compare the differences in immune cell populations and immune functions between the high-risk and low-risk groups within the training cohort. This analysis facilitated a deeper understanding of the immune landscape in relation to the risk stratification of EC patients.

### 2.10 Immune infiltration cell correlation analysis

The CIBERSORT software was utilized to derive the relative abundance of 22 immune cell types from the EC transcriptome data, employing the e1071, parallel, and preprocessCore R packages (20). Samples with a P>0.05 were excluded from the analysis to ensure the reliability of the infiltrating immune cell data. Subsequently, a correlation analysis was performed to examine the relationships between the 22 infiltrating immune cell types and the differentially expressed ARGs incorporated in the risk prognostic model. This analysis was conducted using the limma, reshape2, and ggpubr R packages, providing insights into the interactions between immune infiltration and ARG expression in the context of EC prognosis.

### 2.11 Differential analysis of immune checkpoints

The differential expression of immune checkpoint-related genes within the risk prognostic models of the training cohort was analyzed using the limma, reshape2, ggplot2, and ggpubr R packages. This analysis identified the immune checkpoint-related genes that exhibited significant differences between the high-risk and low-risk groups, providing valuable insights into the potential role of immune regulation in the prognosis of EC.

### 2.12 Drug sensitivity analysis

Drug sensitivity analysis was performed using the limma, ggpubr, and pRRophetic R packages to identify medications with differential sensitivities in the risk prognostic model of the training cohort, applying a significance threshold of P<0.001. This approach facilitated the identification of potential therapeutic agents for EC by screening for drugs that may offer therapeutic benefits based on the model’s prognostic stratification.

### 2.13 Cell culture

Human EC cell lines, KYSE-30 and KYSE-180, along with normal esophageal epithelial cells (NE-2), were used in this study. The EC cell lines (KYSE-30 and KYSE-180) were cultured in RPMI 1640 medium supplemented with 10% fetal bovine serum (FBS). In contrast, the normal esophageal epithelial cells (NE-2) were cultured in a mixture of Defined Keratinocyte-SFM (DK-SFM) and Epilife medium. All cell cultures were maintained at 37°C in a humidified incubator with 5% CO_2_ to support optimal growth conditions.

### 2.14 Real-time quantitative PCR (RT-qPCR)

Total RNA was extracted from EC cell lines and normal esophageal epithelial cells (NE-2) using TRIzol Reagent (Catalog No. 15596018, Life Technologies Invitrogen), following the manufacturer’s protocol. RNA was subsequently reverse transcribed and amplified using ChamQ Universal SYBR qPCR Master Mix (Cat#: Q711-02, Vazyme), according to the manufacturer’s guidelines. The mRNA expression levels of ARGs were quantified using RT-qPCR. Primer pairs, synthesized by Accurate Biology, are listed in **Table 1**. Gene expression was normalized to the β-actin reference gene, and relative expression levels were calculated using the. method.

**Table 1:** Primer sequences for RT-qPCR.

Genes	Forward	Reverse
β-actin	TGGCACCCAGCACAATGAA	CTAAGTCATAGTCCGCCTAGAAGCA
CDK1	ACAGGTCAAGTGGTAGCCAT	ACCTGGAATCCTGCATAAGCAC
FOXC2	CAACATGTTCGAGAACGGCAG	CTCGCTCTTGATCACCACCTTC
IL17A	ACAACCGATCCACCTCACCTT	TGGTAGTCCACGTTCCCATCAG
MAPK1	CGTTGGTACAGGGCTCCAGAA	CTGCCAGAATGCAGCCTACAGA
OLFM3	ATGACTACGAGGAACTACACCAA	TCATCAGTTTGCCACATGTTAGC
PIP5K1C	GTTCAATCGCTCCGCCTGTC	GATTGTCACGCACCAGACCAC

### 2.15 Statistical analysis

All statistical analyses and visualizations were conducted using R software (version 4.1.2) and GraphPad Prism v9.0.0. Statistical significance was assessed using one-way analysis of variance (ANOVA), with a P <0.05 considered indicative of statistical significance. Each experiment was performed independently a minimum of three times to ensure reproducibility and reliability of the results.

## 3 Results

**Figure 1:**
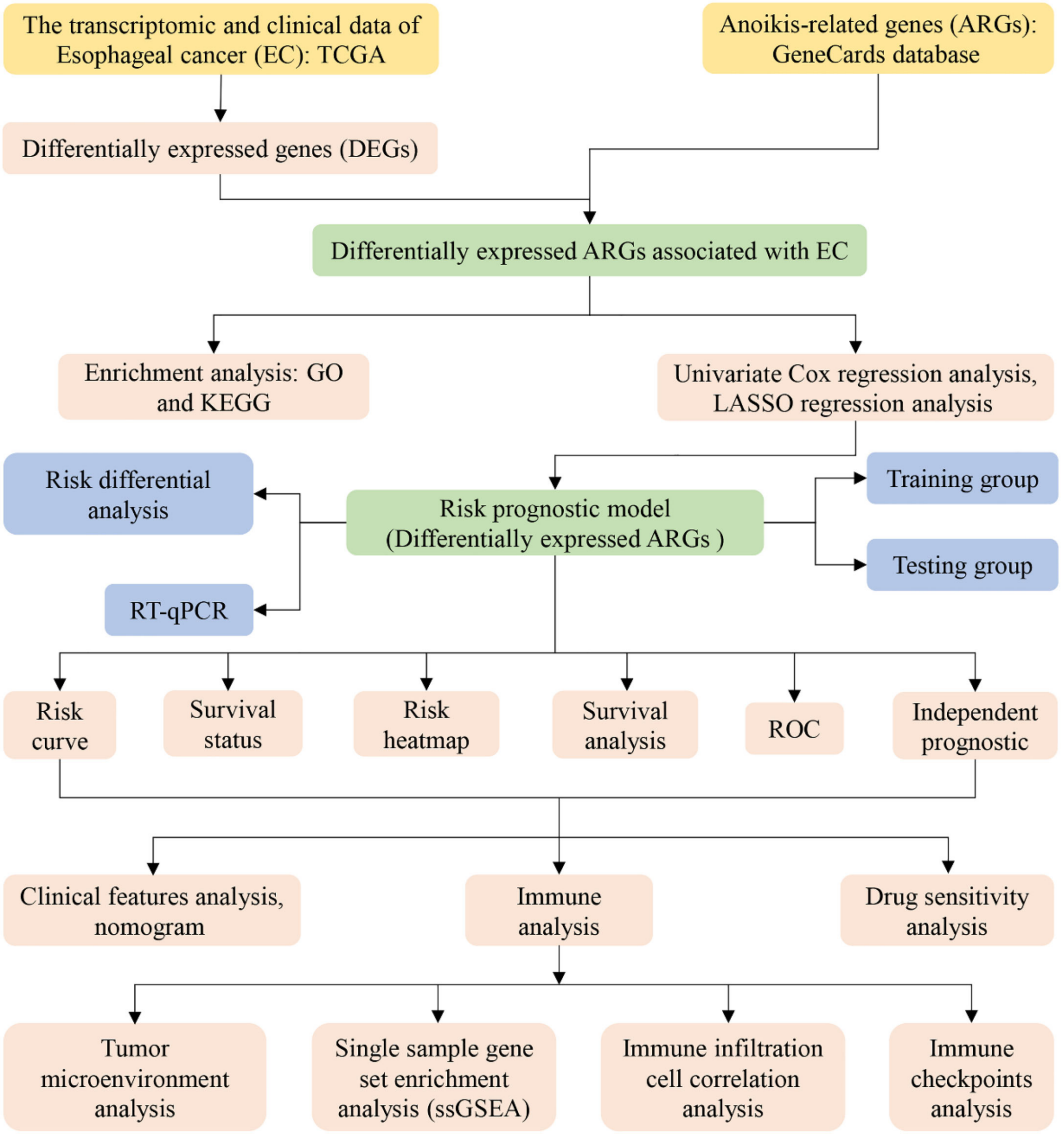
Flow chart of our study.

To enhance the clarity and comprehension of our study, we have provided a flowchart summarizing the key steps of the research, as illustrated in **Figure 1**.

### 3.1 EC-related differentially expressed ARGs

**Figure 2:**
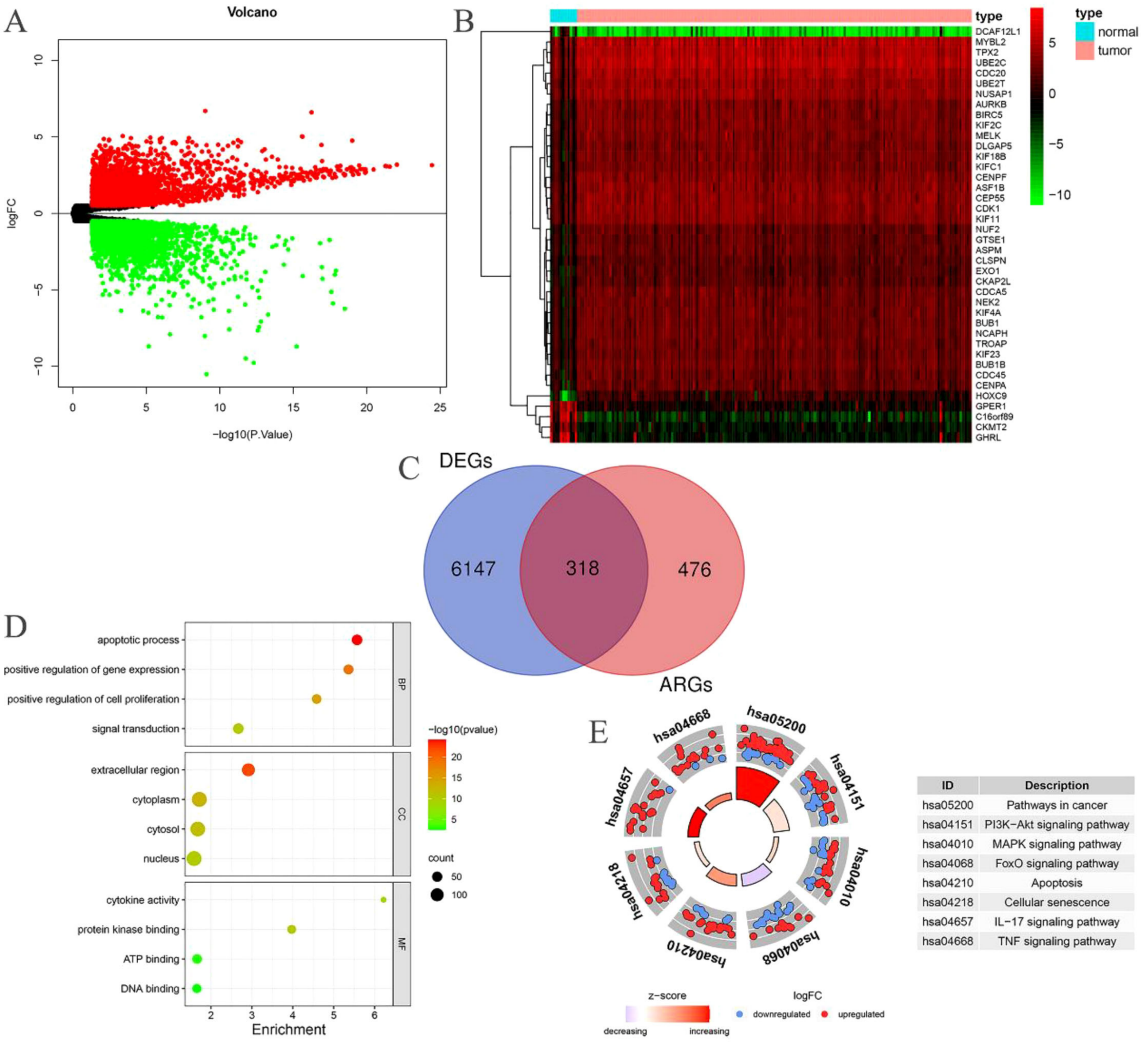
EC-related differentially expressed ARGs. **(A)** Volcano plot of DEGs in patients with EC; **(B)** Heatmap of DEGs in patients with EC; **(C)** The intersection of EC-related ARGs and DEGs results in EC-related differentially expressed ARGs; **(D)** GO enrichment analysis of the EC-related differentially expressed ARGs; **(E)** KEGG enrichment analysis of the EC-related differentially expressed ARGs.

Through the intersection of 794 ARGs with the genes in the EC transcriptome data, we identified 794 EC-related ARGs. Differential expression analysis yielded 6465 DEGs, of which 3133 were upregulated and 3332 were downregulated. The resulting DEGs were visualized using volcano plots (**Figure 2A**) and heatmaps (**Figure 2B**). Upon intersecting the 6465 DEGs with the 794 EC-related ARGs, we identified 318 EC-related differentially expressed ARGs (**Figure 2C**).

### 3.2 Enrichment analysis

The BP category revealed significant enrichment in apoptotic processes, signal transduction, positive regulation of gene expression, and positive regulation of cell proliferation among the 318 EC-related differentially expressed ARGs. In terms of CC, substantial enrichment was observed in the cytoplasm, nucleus, cytosol, and extracellular space. MF related to ATP binding, DNA binding, protein kinase binding, and cytokine activity were also markedly enriched (**Figure 2D**). Additionally, these 318 EC-related differentially expressed ARGs were notably enriched in several cancer-related pathways, including pathways in cancer, PI3K-Akt signaling, MAPK signaling, FoxO signaling, apoptosis, cellular senescence, IL-17 signaling, and TNF signaling (**Figure 2E**).

### 3.3 Construction of risk prognostic model

**Figure 3:**
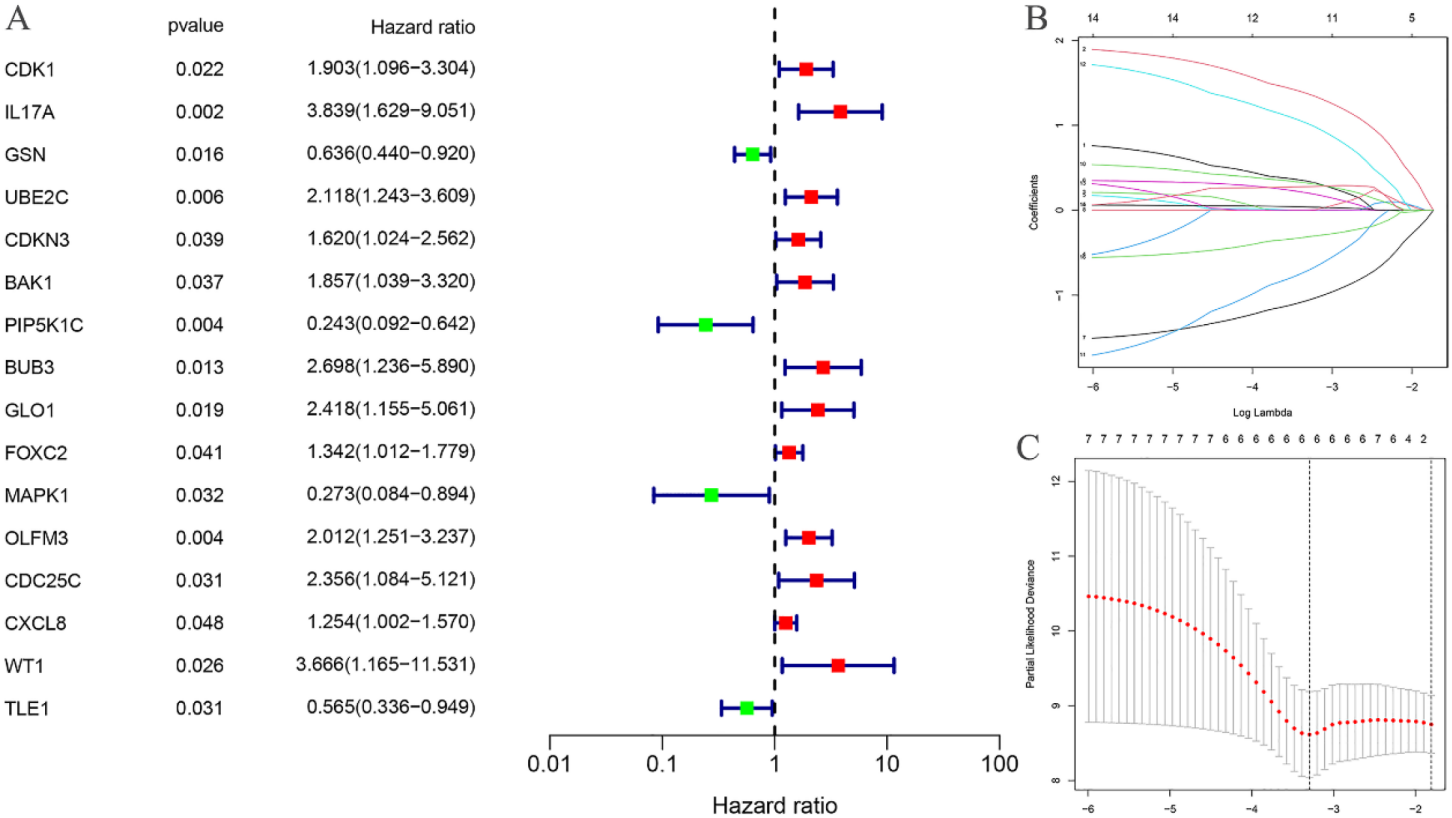
Construction of risk prognostic model. **(A)** Univariate Cox regression analysis obtained 16 candidates prognostic EC-related differentially expressed ARGs; **(B)** Lasso Cox regression analysis; **(C)** Selection of the optimal penalty parameter for Lasso regression.

Univariate Cox regression analysis was performed on the 318 differentially expressed EC-related ARGs to calculate the Hazard Ratio (HR) values. This analysis identified 16 ARGs that were significantly associated with the prognosis of EC (**Figure 3A**). LASSO regression analysis was then employed to determine the optimal number of ARGs for model construction, with a penalty parameter (λ) value revealing that six ARGs were most appropriate for inclusion in the prognostic model (**Figures 3B, C**). Using the constructed model, a riskscore was calculated for each EC sample. Based on the median riskscore, the training cohort was stratified into high-risk (N=40) and low-risk (N=40) groups. Similarly, the testing cohort was divided into high-risk (N=40) and low-risk (N=39) groups according to the median riskscore.

### 3.4 Validation of the risk prognostic model

**Figure 4:**
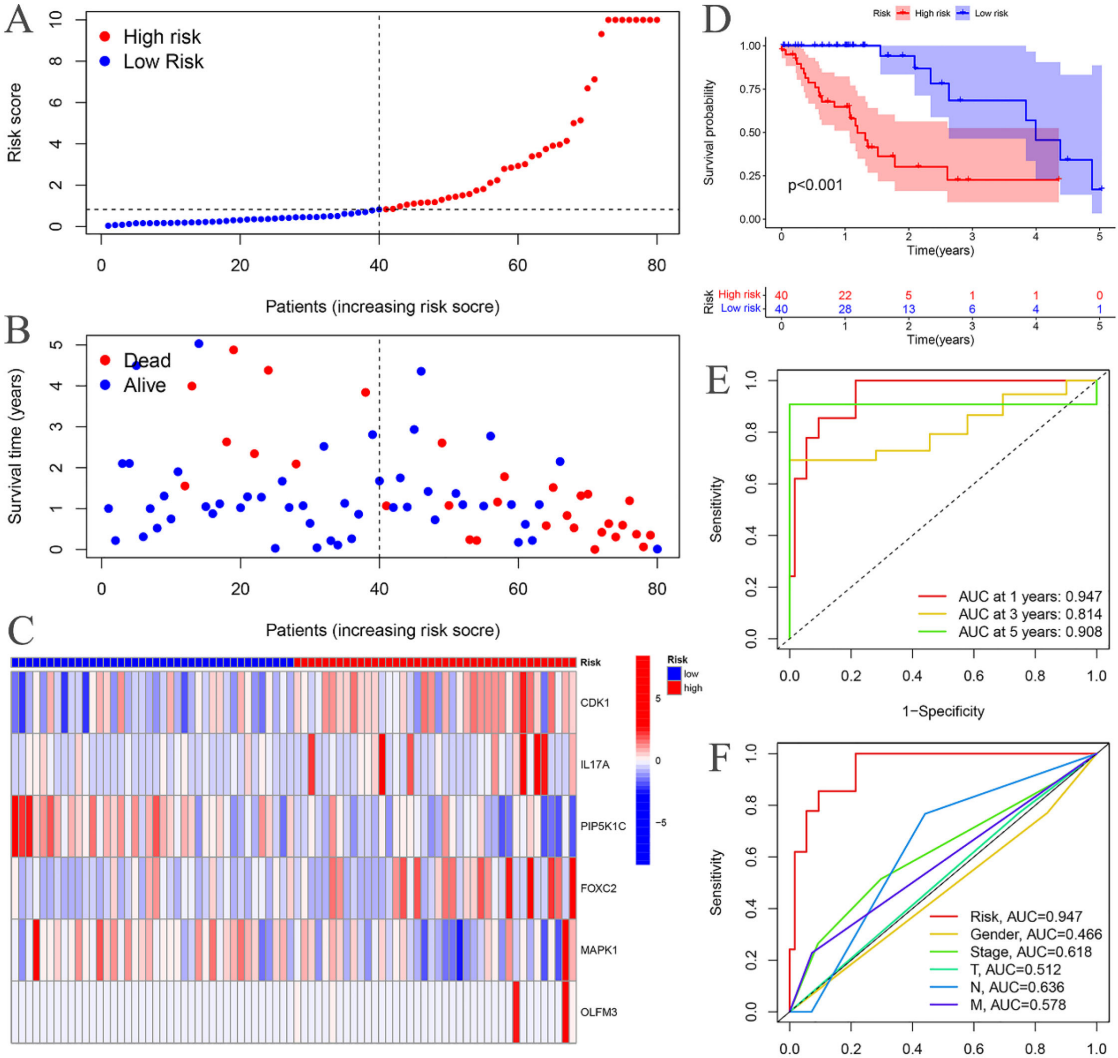
Training groups. **(A)** Risk curve. **(B)** Survival status map; **(C)** Risk heatmap; **(D)** Survival curve. **(E)** Time-dependent ROC curves; **(F)** ROC curves for the riskScore and clinical features.

**Figure 5:**
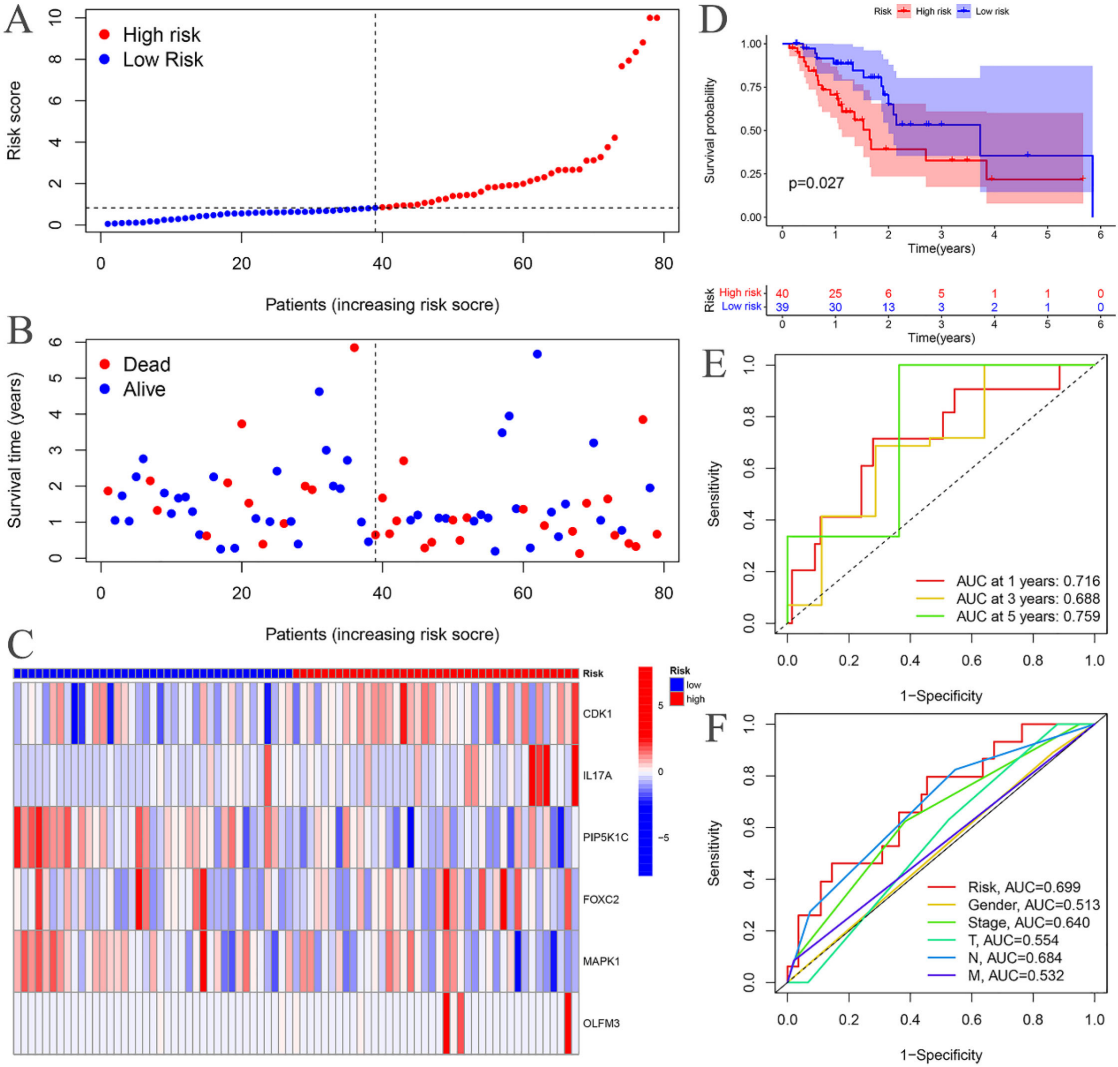
Testing groups. **(A)** Risk curve. **(B)** Survival status map; **(C)** Risk heatmap; **(D)** Survival curve. **(E)** Time-dependent ROC curves; **(F)** ROC curves for the riskScore and clinical features.

**Figure 6:**
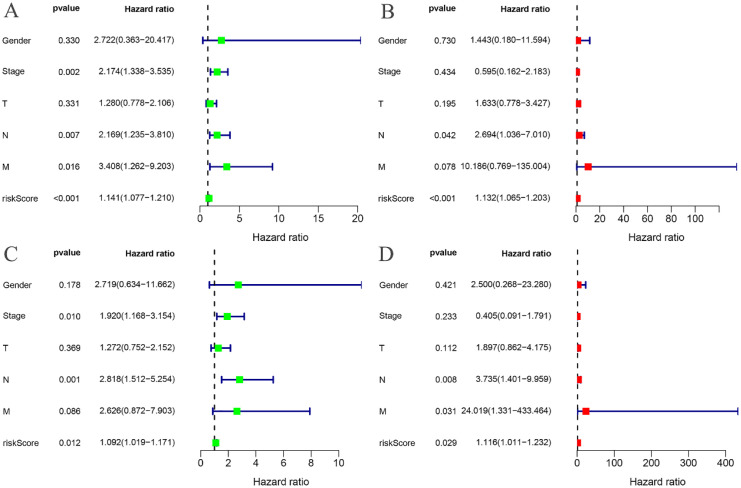
Independent prognostic analysis. **(A)** Univariate COX regression analysis of the training groups; **(B)** Multivariate COX regression analysis of the training groups; **(C)** Univariate COX regression analysis of the testing groups; **(D)** Multivariate COX regression analysis of the testing groups.

The risk curves for both the training and testing groups demonstrate a consistent increase in the risk of EC as patients progress from the low-risk to the high-risk group (**Figures 4A**, **5A**). Correspondingly, survival status maps for both groups indicate a higher mortality rate in patients with EC as the risk increases (**Figures 4B**, **5B**). According to the risk heatmaps of both groups, the expression levels of high-risk ARGs, including CDK1, IL17A, FOXC2, and OLFM3, progressively rise from the low-risk to the high-risk group, while the expression levels of low-risk ARGs, PIP5K1C and MAPK1, decrease accordingly (**Figures 4C**, **5C**). Survival analysis for both the training and testing groups reveals significant differences in patient survival between the high- and low-risk groups (**Figures 4D**, **5D**). Time-dependent ROC curves for both groups demonstrate a higher area under the curve (AUC) at 1, 3, and 5 years (**Figures 4E**, **5E**). ROC curves for the risk score and clinical features in both groups also show that the risk score, pathological stage, and N stage have larger AUC (**Figures 4F**, **5F**). In the training group, univariate independent prognostic analysis identified that the risk score, pathological stage, N, and M stages can all serve as independent prognostic indicators (**Figure 6A**). Multivariate independent prognostic analysis in the training group further revealed that the risk score and N stage are significant independent prognostic factors (**Figure 6B**). In the testing group, univariate analysis also identified risk score, pathological stage, and N stage as independent prognostic factors (**Figure 6C**), and multivariate analysis confirmed that risk score, N, and M stages are independent prognostic indicators (**Figure 6D**). A comprehensive analysis of these independent prognostic factors suggests that both the risk score from the prognostic model and N stage may serve as reliable independent prognostic indicators for EC patients.

### 3.5 Risk differential analysis

**Figure 7:**
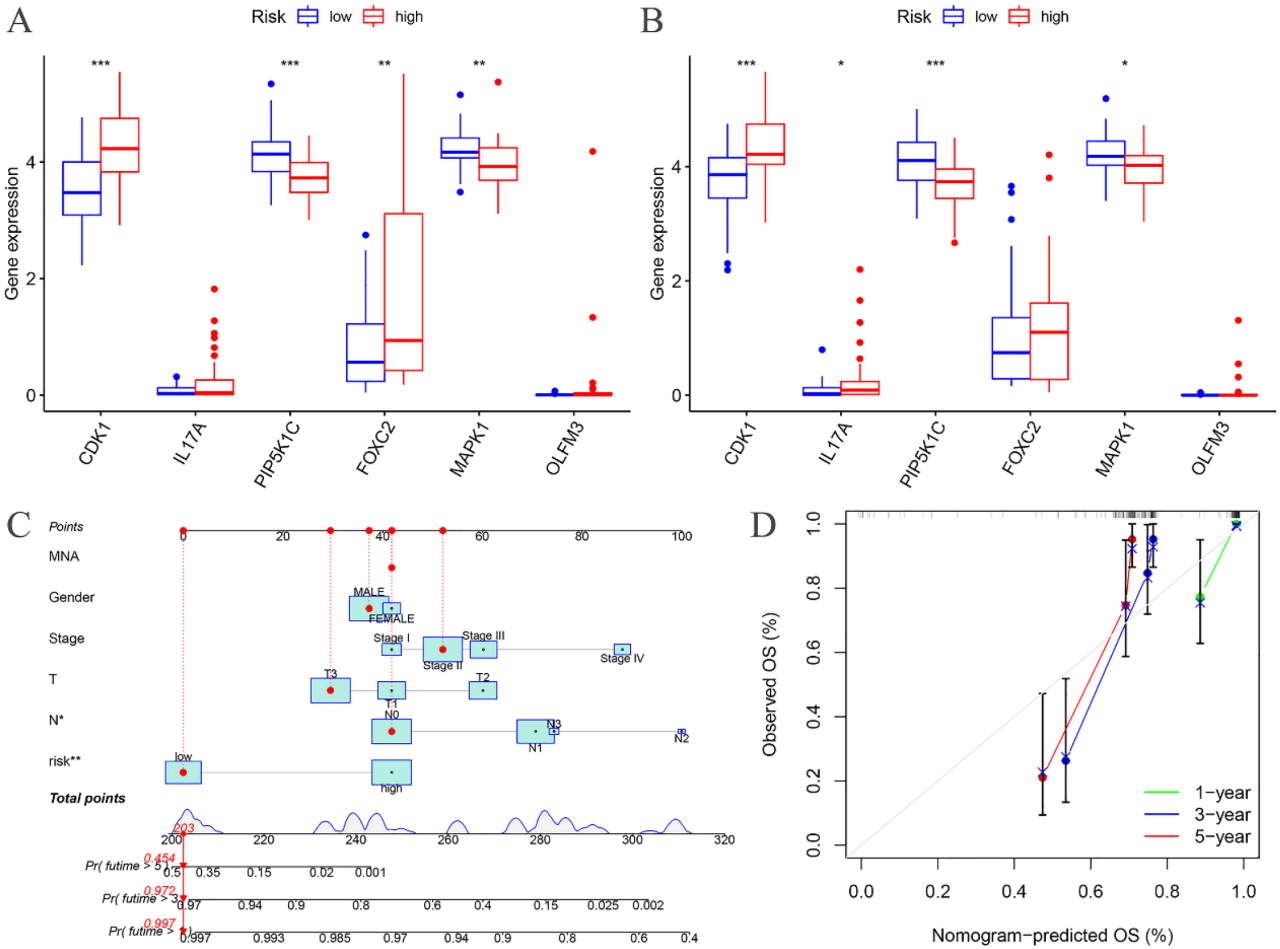
Risk differential analysis and nomogram. **(A)** Risk differential analysis of the training groups; **(B)** Risk differential analysis of the testing groups; **(C)** Nomogram of the training groups; **(D)** Calibration curve.

The expression levels of CDK1, PIP5K1C, FOXC2, and MAPK1 were found to differ significantly between the high- and low-risk groups in the training cohort (**Figure 7A**). Similarly, in the testing cohort, CDK1, IL17A, PIP5K1C, and MAPK1 exhibited notable differences in expression between the high- and low-risk groups (**Figure 7B**).

### 3.6 Clinical features analysis

**Figure 8:**
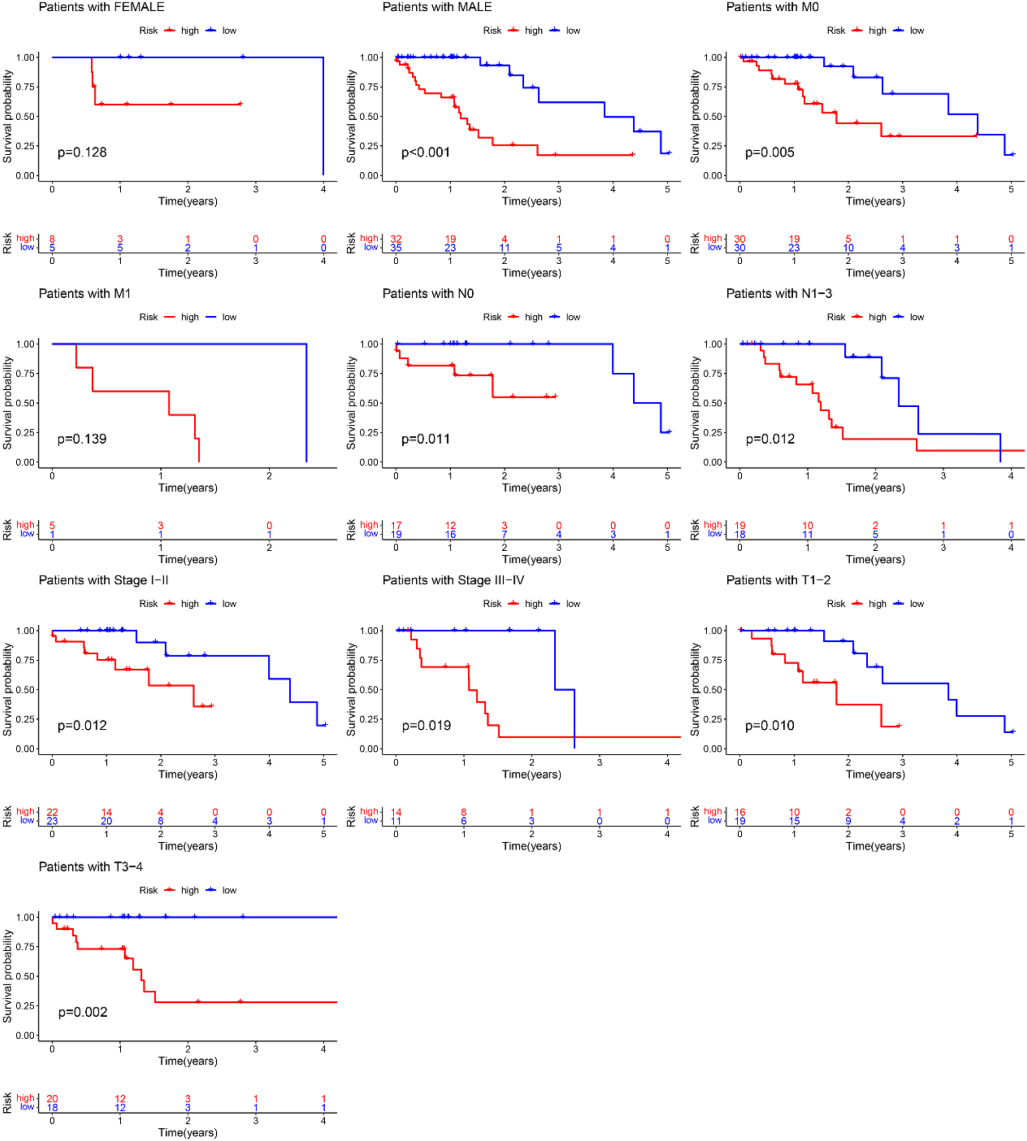
Training groups, clinical validation of the risk prognosis model.

A nomogram model was developed based on the riskscore and clinical features to predict the 1-, 3-, and 5-year survival rates of EC patients (**Figure 7C**). The calibration curve closely aligned with the ideal reference curve (represented by the gray straight line), indicating a high degree of concordance between the predicted and observed survival outcomes (**Figure 7D**). This finding underscores the strong predictive accuracy of the nomogram model for EC patient prognosis. Further model validation within various clinical subgroups revealed that the risk prognostic model performed well across the stage, T, and N clinical groupings. Additionally, the model was effective for male patients in the gender subgroup and for patients with M0 stage in the M clinical subgroup (**Figure 8**).

### 3.7 Tumor microenvironment analysis

**Figure 9:**
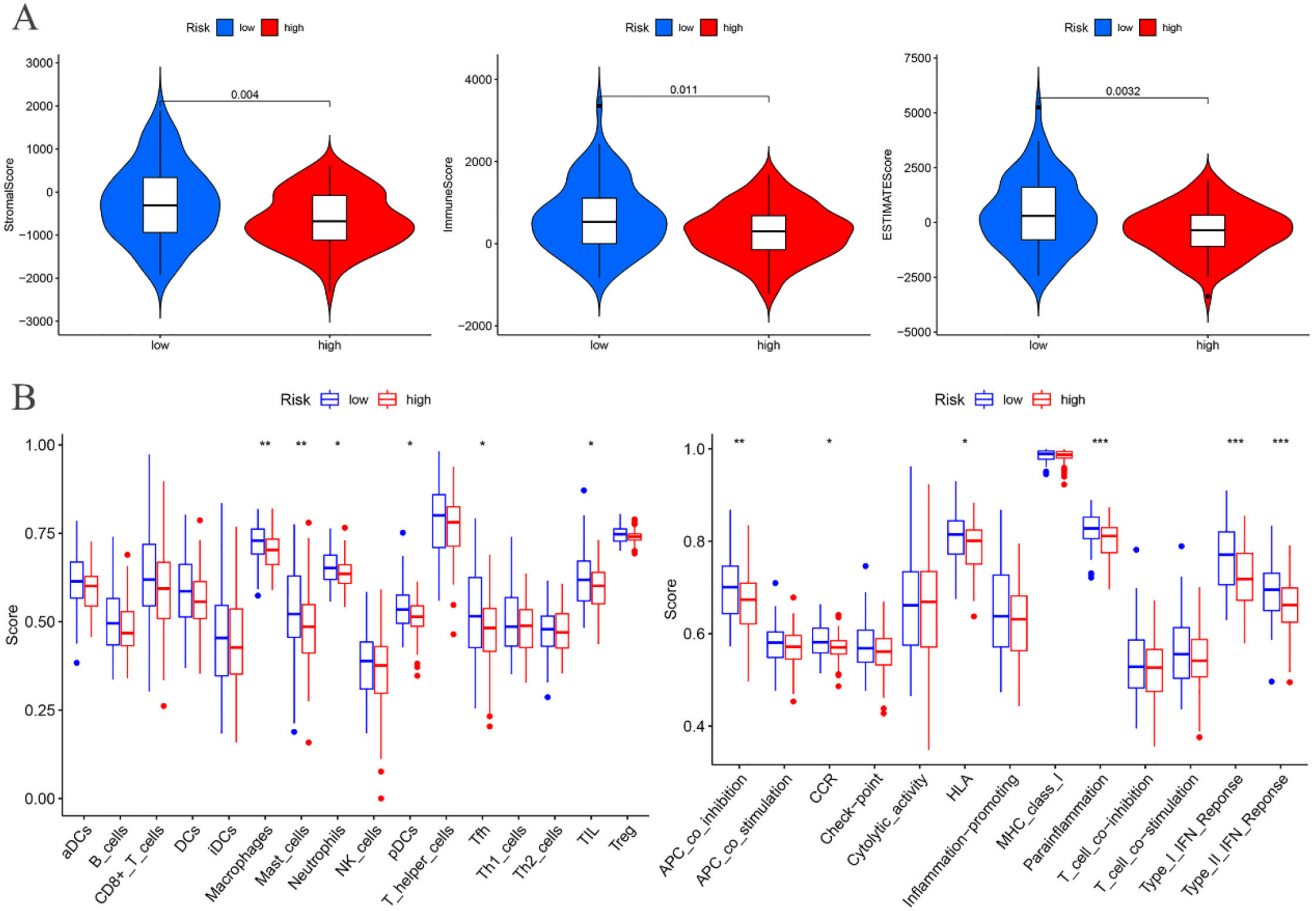
Training groups. **(A)** Differential analysis of tumor microenvironment. **(B)** Immune cell and Immune function difference analysis of ssGSEA.

The tumor microenvironment analysis of the risk prognostic model in the training group revealed significant differences in the stromalScores, immuneScores, and ESTIMATEScores between the high- and low-risk groups. Notably, the scores in the low-risk group were higher compared to those in the high-risk group (**Figure 9A**).

### 3.8 ssGSEA

Differential analysis of immune cell populations in the risk prognostic model of the training group revealed a significant downregulation of macrophages and mast cells in the high-risk group (P < 0.01). Furthermore, immune function analysis indicated a notable downregulation of pathways, including APC co-inhibition, parainflammation, Type I IFN response, and Type II IFN response, in the high-risk group (P < 0.01) (**Figure 9B**).

### 3.9 Immune infiltration cell correlation analysis

**Figure 10:**
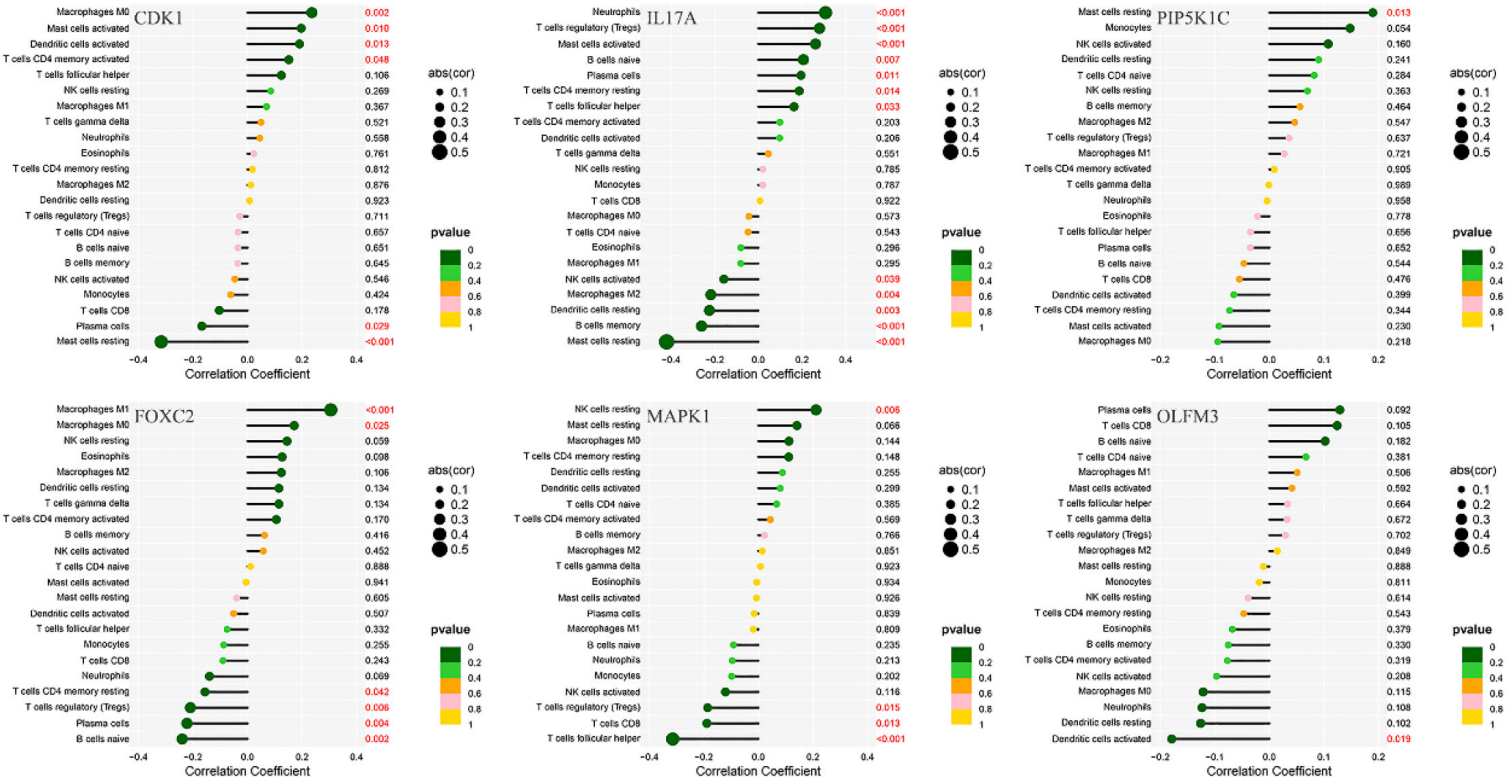
Correlation analysis between immune infiltration cells and differential ARGs involved in model construction.

Immunological correlation analysis revealed that CDK1 exhibited a negative correlation with resting mast cells and plasma cells, while it was positively associated with activated CD4 memory T cells, activated dendritic cells, activated mast cells, and M0 macrophages. Additionally, resting mast cells, memory B cells, resting dendritic cells, M2 macrophages, and activated NK cells showed a negative correlation with IL17A, whereas plasma cells, naïve B cells, regulatory T cells (Tregs), and neutrophils were positively correlated with IL17A. PIP5K1C demonstrated a positive correlation with resting mast cells. FOXC2 was positively correlated with M0 and M1 macrophages but negatively correlated with naïve B cells, plasma cells, Tregs, and resting CD4 T cells. MAPK1 showed a positive correlation with resting NK cells and a negative correlation with follicular helper T cells, CD8 T cells, and Tregs. Finally, OLFM3 exhibited a negative correlation with activated dendritic cells (**Figure 10**).

### 3.10 Differential analysis of immune checkpoints

**Figure 11:**
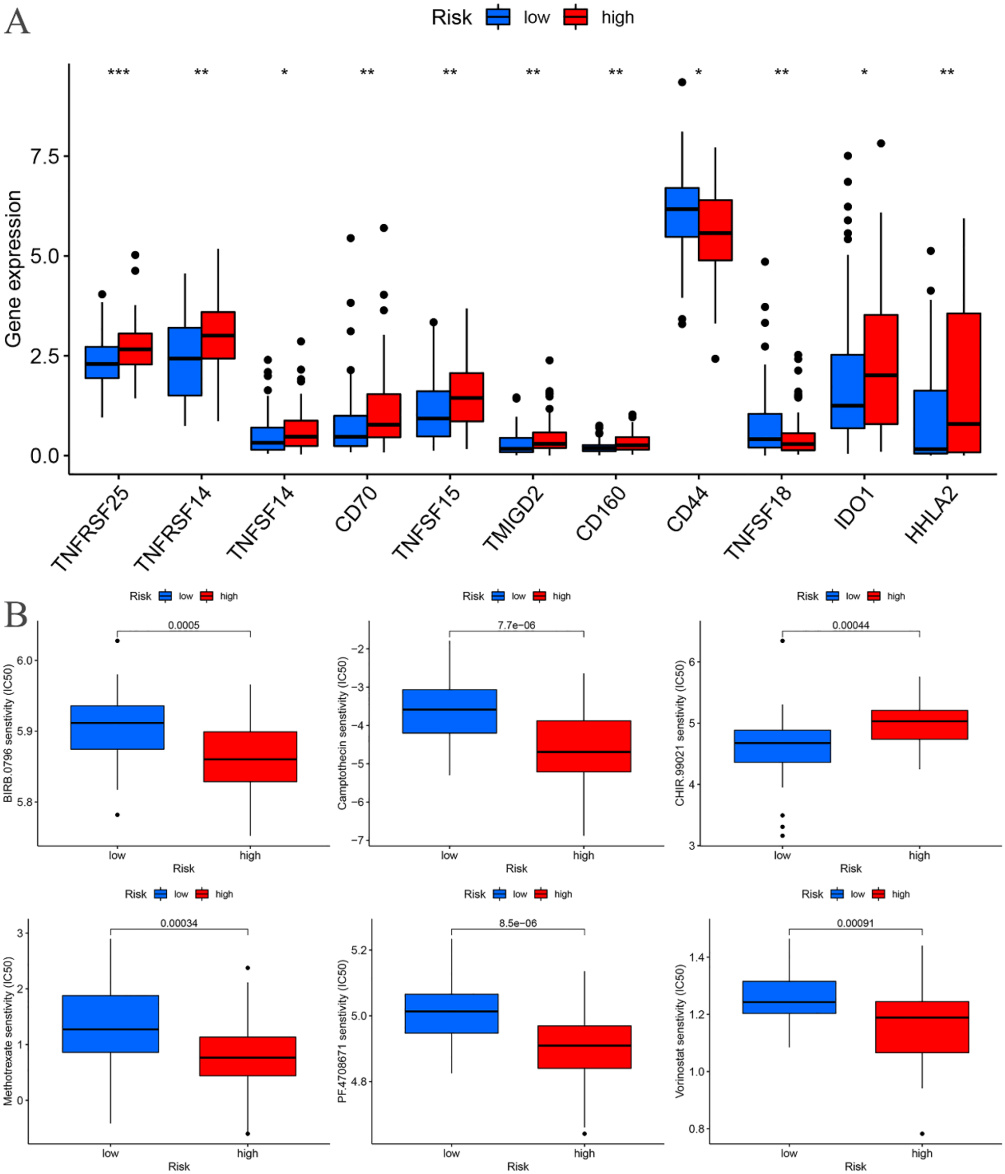
Training cohort. **(A)** Differential analysis of immune checkpoints. **(B)** Drug sensitivity analysis. *p < 0.05, **p < 0.01, ***p < 0.001.

Immune checkpoint differential analysis revealed significant differences in the expression of 11 immune checkpoint-related genes between the high-risk and low-risk groups in the training cohort. Among these, TNFRSF25, TNFRSF14, CD70, TNFSF15, TMIGD2, CD160, TNFSF18, and HHLA2 exhibited exceptionally high statistical significance (P < 0.01) (**Figure 11A**).

### 3.11 Drug sensitivity analysis

A drug sensitivity analysis of the risk prognostic model in the training cohort revealed that several compounds, including BIRB.0796, Camptothecin, CHIR.99021, Methotrexate, PF.4708671, and Vorinostat, demonstrated statistically significant sensitivity across both high-risk and low-risk groups (P < 0.001). Notably, patients in the high-risk group exhibited heightened sensitivity to BIRB.0796, Camptothecin, Methotrexate, PF.4708671, and Vorinostat, whereas those in the low-risk group showed increased responsiveness to CHIR.99021 (**Figure 11B**).

### 3.12 Validation of the expression of ARGs in EC

**Figure 12:**
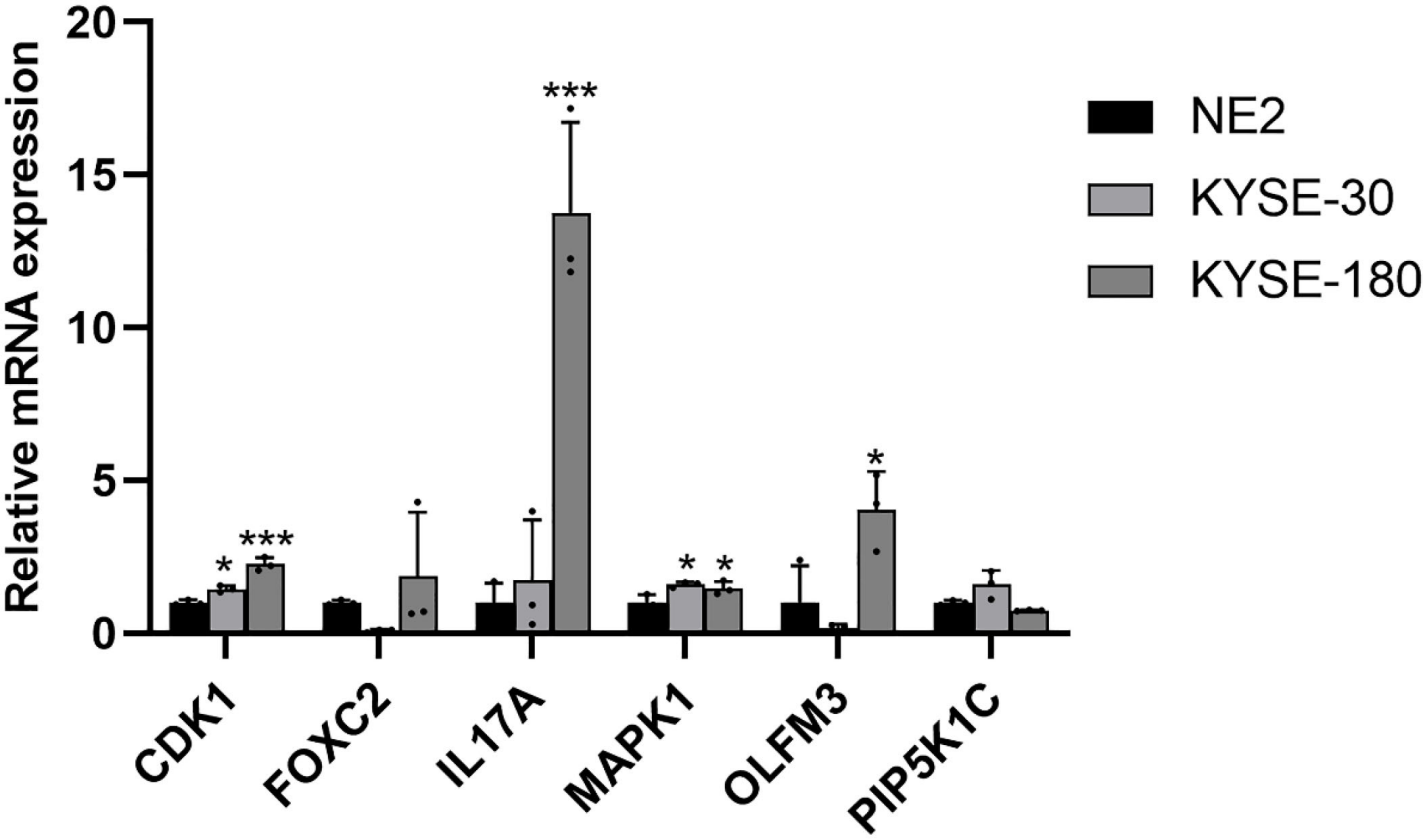
Validation of the mRNA expression level of ARGs in EC cell lines. *p < 0.05, ***p < 0.001, each experiment was repeated three times.

To further assess the expression of ARGs in EC, mRNA expression levels were analyzed in two EC cell lines, KYSE-30 and KYSE-180, with normal esophageal epithelial cells (NE-2) serving as the control group. Compared to NE-2 cells, the mRNA levels of CDK1 and MAPK1 were notably upregulated in both EC cell lines, KYSE-30 and KYSE-180. Additionally, the mRNA expression of FOXC2, IL17A, and OLFM3 was significantly elevated in the KYSE-180 cell line when compared to the control group (**Figure 12**).

## 4 Discussion

In this study, we developed an innovative risk prognostic model based on ARGs, which effectively predicts the survival outcomes and characterizes the immunological microenvironment of EC patients. This model is capable of forecasting overall survival in EC patients, with N serving as an independent prognostic indicator. The model incorporates six ARGs: PIP5K1C and MAPK1, which are identified as low-risk ARGs, and CDK1, IL17A, FOXC2, and OLFM3, which are high-risk ARGs for EC patients. Additionally, we constructed a nomogram model that significantly enhances the prediction of EC patient prognosis. Our findings also revealed that the immuneScores of high-risk EC patients were lower compared to low-risk patients. Furthermore, immune cells, such as macrophages and mast cells, were notably downregulated in the high-risk group. Immune functions related to APC co-inhibition, parainflammation, Type I IFN response, and Type II IFN response were also significantly reduced in the high-risk group. We identified eight immune checkpoint-related genes—TNFRSF25, TNFRSF14, CD70, TNFSF15, TMIGD2, CD160, TNFSF18, and HHLA2—that demonstrated high statistical significance. Lastly, we identified six drugs—BIRB.0796, Camptothecin, CHIR.99021, Methotrexate, PF.4708671, and Vorinostat—that may hold potential therapeutic value for EC patients.

CDK1, a key regulator of the eukaryotic cell cycle, orchestrates crucial processes such as the centrosome cycle and the initiation of mitosis (21, 22). As a therapeutic target, CDK1 has gained significant attention for its potential in cancer treatment, with inhibitors being particularly promising (22). In pancreatic cancer, CDK1 inhibition has been shown to overcome IFNG-mediated adaptive immune resistance (23). In melanoma, CDK1 collaborates with Sox2 to promote tumor initiation (24), while in gastrointestinal stromal tumors, CDK1 emerges as a novel vulnerability independent of the cell cycle, offering potential therapeutic avenues for advanced-stage patients (25). NCAPG-driven CDK1 facilitates the malignant progression of non-small cell lung cancer through ERK signaling activation (26). In prostate cancer, SLC14A1 downregulation enhances CDK1/CCNB1 and mTOR pathway activity, accelerating tumorigenesis (27). Conversely, NFIX inhibits breast cancer cell proliferation by delaying mitotic entry via CDK1 suppression (28). Additionally, CDK1 exerts a proapoptotic function, sensitizing ovarian cancer cells to paclitaxel and overcoming resistance when co-administered with duloxetine (29). FOXC2, a transcription factor belonging to the forkhead/winged helix family, is essential for embryonic development and organogenesis (30). As a critical regulator of tumor progression, FOXC2 has become a valuable biomarker for predicting cancer aggressiveness and patient prognosis (31, 32). For instance, in esophageal squamous cell carcinoma (ESCC) (33) and hepatocellular carcinoma (34), FOXC2 serves as a prognostic marker, playing a role in tumor growth and invasion (35). Moreover, FOXC2 promotes chemoresistance in nasopharyngeal cancer by inducing epithelial-mesenchymal transition, while also modulating the YAP signaling pathway and enhancing glycolysis (35). FOXC2 serves as a prognostic biomarker and facilitates tumor growth and invasive potential in hepatocellular carcinoma (34). In ovarian cancer, stanniocalcin 1 enhances metastatic capacity, lipid metabolism, and cisplatin resistance through the FOXC2/ITGB6 signaling pathway (36). Furthermore, MRTX1133 suppresses progression of KRAS G12D-mutated colorectal cancer by inducing ferroptosis via the METTL14/LINC02159/FOXC2 axis (37).

PIP5K1C contributes to the formation of cell junctions and is involved in growth factor-induced directional cell migration and adhesion. It also regulates the establishment of adherens junctions by facilitating the trafficking of CDH1/cadherin (38). Genetic variations in PIP5K1C and MVB12B, which are part of the endosome-related pathway, have been linked to survival outcomes in cutaneous melanoma (39). MiR-4649-5p functions as a tumor suppressor in triple-negative breast cancer through direct targeting of PIP5K1C, consequently enhancing the growth-inhibitory efficacy of the AKT inhibitor capivasertib (40). Furthermore, genetic variants in PIP5K1C and MVB12B, components of the endosomal pathway, have been identified as novel prognostic markers for cutaneous melanoma-specific survival (39). Functioning as a critical signaling node, MAPK1 integrates diverse biochemical stimuli to regulate fundamental cellular processes including proliferation, differentiation, transcriptional modulation, and developmental programs. The enzymatic activation of this kinase is contingent upon phosphorylation by upstream regulators. Following activation, MAPK1 undergoes nuclear translocation where it mediates phosphorylation of nuclear substrates. MAPK1 has been shown to correlate with overall survival in EC patients (41). MicroRNA-490-3p downregulates MAPK1, inhibiting ESCC cell growth and promoting apoptosis (41). MiR-574-3p exerts tumor-suppressive effects in esophageal cancer by directly targeting FAM3C and MAPK1, thereby inhibiting cellular proliferation and invasive potential (42). Furthermore, MAPK1/3 regulates hepatic lipid metabolism via ATG7-dependent autophagy (43). LINC00511 drives cervical cancer progression through modulation of the miR-497-5p/MAPK1 regulatory axis (44). Independent systems-level analysis of ARGs has identified MAPK1 as a potential therapeutic target for osteosarcoma patients receiving neoadjuvant chemotherapy (45). IL-17A plays a pivotal role in host defense by modulating immune responses, facilitating leukocyte recruitment, and promoting tissue repair, particularly through the activation of innate immunity. Furthermore, IL-17A acts on non-hematopoietic cells to enhance chemokine secretion, thereby recruiting myeloid cells to sites of inflammation. Heterozygosity at the IL7A-197 A/G locus confers protection against both the onset and severity of colorectal cancer within the Bulgarian cohort (46). Furthermore, P2X7 receptor activation modulates the reinforcing and psychomotor effects of METH, possibly via an IL-17A-dependent mechanism, given this cytokine’s emerging role in anxiety regulation (47).

Despite the absence of prior reports on OLFM3, our findings establish its significant prognostic value in conjunction with other critical molecular markers. The six ARGs identified in this investigation - PIP5K1C, MAPK1, CDK1, IL17A, FOXC2, and OLFM3 - collectively represent a novel molecular signature with robust potential for predicting clinical outcomes in EC patients. These results not only reveal previously unappreciated roles for these ARGs in EC pathogenesis but also suggest complex interactions within anoikis-related pathways that warrant detailed mechanistic exploration. Future studies should focus on elucidating the precise molecular networks through which these biomarkers influence disease progression and treatment response.

Tumor-associated macrophages (TAMs), which are among the most prevalent immune cells in the tumor microenvironment, play a crucial role in mediating tumor initiation and progression (48). The infiltration of CD68+/CD163- macrophages has been identified as a poor prognostic factor following neoadjuvant chemotherapy in EC and gastric adenocarcinoma (49). Additionally, high levels of M2 macrophage infiltration in ESCC have been associated with unfavorable prognosis and suboptimal pathological responses to neoadjuvant therapy (3). Mast cells, multifunctional immune cells primarily located in the skin, respiratory mucosa, and gastrointestinal tract, are also implicated in cancer progression (50). Elevated mast cell density correlates with tumor growth in ESCC, and is positively associated with tumor angiogenesis, invasion depth, lymph node metastasis, and overall tumor progression (51, 52), all of which contribute to poor prognosis in ESCC patients (52). Moreover, research has shown that the number of activated CD169 macrophages and effector CD8 T cells within the same region is positively correlated with a subset of mast cells capable of producing IL-17 in the esophageal muscular propria, as opposed to the tumor nests, suggesting a favorable prognosis and improved survival (53). In this study, we observed that the levels of macrophages and mast cells were significantly reduced in the high-risk group, along with a marked downregulation of immune functions, including APC co-inhibition, parainflammation, and both Type I and Type II IFN responses.

One potential therapeutic strategy for EC involves the inhibition of immune checkpoint proteins (54). Previous studies have demonstrated that peripheral T lymphocytes in EC patients exhibit dysregulated expression of CD160 (53). In addition, TNFSF18 has been found to be significantly correlated with clinical factors such as gender, TNM stage, and survival outcomes in EC patients (p < 0.05) (55). HHLA2, a recently identified member of the B7 family of immune checkpoints, has been shown to be highly expressed in lung cancer (56) and colorectal carcinoma (57). Furthermore, elevated HHLA2 expression in ESCC, clear cell renal cell carcinoma, and pancreatic dual adenocarcinoma has been associated with better prognosis (58, 59). These findings suggest that HHLA2 could serve as a valuable biomarker across multiple cancer types. However, the roles of other immune checkpoint-related genes, such as TNFRSF25, TNFRSF14, CD70, TNFSF15, and TMIGD2, in EC remain largely unexplored. The findings from this study emphasize the potential importance of these immune checkpoints in EC immunotherapy. Future investigations should focus on elucidating their expression profiles in EC tissues, delineating their mechanistic contributions to disease progression, and evaluating their utility as therapeutic targets or biomarkers for immunotherapy interventions in EC.

BIRB.0796, a highly potent inhibitor of p38, has shown promise as a therapeutic agent (60). Camptothecin, a well-established anticancer drug, induces apoptosis and autophagy in cancer cells (61). In EC cells, camptothecin inhibits neddylation, thereby triggering protective autophagy through the NF-κB/AMPK/mTOR/ULK1 signaling axis (61). CHIR99021, a GSK-3β inhibitor, is involved in Wnt pathway signaling (62). Similar to Wnt, CHIR99021 suppresses GSK-3 activity, potentially activating β-catenin-Lef/Tcf signaling, which is essential for maintaining cancer stem cells (62, 63). Methotrexate, a widely used chemotherapeutic and immunosuppressive agent, acts by inhibiting dihydrofolate reductase, thereby preventing the conversion of dihydrobiopterin (BH2) to tetrahydrobiopterin (BH4). This results in uncoupled nitric oxide synthase activity and sensitization of T cells to apoptosis, reducing immune responses (64, 65). PF-4708671, a selective S6K1 inhibitor, modestly decreases S6 phosphorylation while paradoxically activating the PI3K pathway (66, 67). Its metabolic benefits in muscle and liver cells are attributed to the inhibition of mitochondrial complex I (68). In early cerebral ischemia-reperfusion injury, PF-4708671’s inhibition of p70 ribosomal S6K1 reduced infarct size and vascular permeability (69). Vorinostat, a pan-histone deacetylase inhibitor, has multiple therapeutic applications. It suppresses productive HPV-18 DNA amplification and selectively targets drug-resistant tumor cells (70). Additionally, vorinostat enhances the efficiency of CRISPR-mediated homology-directed repair in human induced pluripotent stem cells (71), making it a versatile candidate for next-generation cancer therapies (72). Camptothecin, a potent topoisomerase I inhibitor, has demonstrated significant clinical efficacy in the treatment of a diverse range of malignancies, including primary liver cancer, gastric cancer, bladder cancer, rectal cancer, head and neck epithelial carcinoma, and leukemia, among others. Similarly, methotrexate, a well-established antifolate agent, has been widely employed in clinical settings for the management of various hematologic and solid tumors, such as acute lymphoblastic leukemia, head and neck squamous cell carcinoma, non-small cell lung cancer, and gynecological malignancies including ovarian and cervical cancers. Vorinostat, a histone deacetylase inhibitor, has been primarily indicated for the treatment of primary cutaneous T-cell lymphoma. While small-molecule kinase inhibitors such as BIRB-0796, CHIR-99021, and PF-470867 have not yet been established as first-line clinical therapeutics, studies suggest their considerable therapeutic potential in many pathological conditions, including EC. Further investigation into their pharmacokinetic profiles, safety, and efficacy in human trials may pave the way for their future clinical application.

Despite the valuable insights provided by this study, certain limitations remain. First, the relatively small sample size of tumor specimens may have affected the statistical power and external generalizability of the findings. Consequently, future studies are warranted to validate these results in larger and more diverse patient cohorts, thereby enhancing the robustness and reproducibility of the conclusions. Expanding the sample size would not only improve the statistical precision of the prognostic model but also strengthen its applicability across different patient populations. Furthermore, this study lacks a validation model for external datasets. Future research should be able to conduct further external verification to enhance generalization. Finally, although this study identified key ARGs associated with EC prognosis and immune regulation, the experimental validation is relatively limited, their specific biological functions and underlying mechanisms remain insufficiently explored, which is an important limitation. Comprehensive functional analyses are needed to elucidate how these ARGs contribute to tumor progression, immune microenvironment modulation, and therapeutic resistance, thereby providing deeper insights into their potential as biomarkers or therapeutic targets. Addressing these limitations in future research will be essential for increasing the translational potential of the findings and advancing the scientific understanding of EC.

## 5 Conclusion

In conclusion, this study successfully established a novel risk prognostic model based on six ARGs, offering a valuable tool for stratifying EC patients according to survival outcomes and immune microenvironment characteristics. The model not only provides insights into the complex interactions between anoikis and EC but also enhances the ability to predict prognosis with high accuracy. Furthermore, a complementary nomogram model was constructed, demonstrating robust predictive capability for long-term survival in EC patients. Additionally, this research identified six candidate therapeutic agents—BIRB.0796, Camptothecin, CHIR99021, Methotrexate, PF-4708671, and Vorinostat—that exhibit sensitivity in EC patients and hold promise for improving clinical outcomes. These findings underscore the potential of personalized medicine approaches in optimizing therapeutic strategies for EC. By integrating gene transcriptomics, immunological, and pharmacological insights, this study lays the groundwork for developing more targeted treatment modalities. The risk model and its associated findings represent a step forward in the pursuit of precision oncology for EC, providing clinicians with actionable tools to enhance survival rates and therapeutic efficacy. Future investigations should focus on validating these models in larger, independent cohorts and exploring the mechanistic roles of the identified ARGs and therapeutic agents to further refine their clinical applicability.

## Data Availability

Publicly available datasets were analyzed in this study. This data can be found here: https://portal.gdc.cancer.gov/.
