# Supplementary material for: Characterization of novel anoikis-related genes as prognostic biomarkers and key determinants of the immune microenvironment in esophageal cancer
Source: Front Immunol. 2025 Jul 11;16:1599171. doi: 10.3389/fimmu.2025.1599171 (PMC12311635; doi:10.3389/fimmu.2025.1599171)
Supplement: Supplementary file 1 [file Table1.docx]

1. **The expression of ARGs was extracted**

library(limma)

expFile="mRNA.txt"

geneFile="gene.txt"

setwd("")

rt=read.table(expFile, header=T, sep="\t", check.names=F)

rt=as.matrix(rt)

rownames(rt)=rt[,1]

exp=rt[,2:ncol(rt)]

dimnames=list(rownames(exp),colnames(exp))

data=matrix(as.numeric(as.matrix(exp)),nrow=nrow(exp),dimnames=dimnames)

data=avereps(data)

data=data[rowMeans(data)>0,]

gene=read.table(geneFile, header=F, check.names=F, sep="\t")

sameGene=intersect(as.vector(gene[,1]), rownames(data))

geneExp=data[sameGene,]

out=rbind(ID=colnames(geneExp),geneExp)

write.table(out,file=" ",sep="\t",quote=F,col.names=F)

1. **Differential analysis**

setwd("")

library(limma)

library("impute")

geo_data<-read.table("geneMatrix.txt",sep="\t",header=T)

geo_data<-as.matrix(geo_data)

rownames(geo_data)=geo_data[,1]

geo_exp<-geo_data[,2:ncol(geo_data)]

dimnames<-list(rownames(geo_exp),colnames(geo_exp))

geo_exp<-matrix(as.numeric(as.matrix(geo_exp)),nrow=nrow(geo_exp),dimnames=dimnames)

mat=impute.knn(geo_exp)

geo_data=mat$data

geo_data=avereps(geo_data)

pdf(file="raw_box.pdf")

boxplot(geo_data,col = "green",xaxt = "n",outline = F)

dev.off()

geo_data=normalizeBetweenArrays(as.matrix(geo_data))

pdf(file="normal_box.pdf")

boxplot(geo_data,col = "red",xaxt = "n",outline = F)

dev.off()

#geo_data=log2(geo_data)

class <- c(rep("normal",3),rep("treatment",3))

design <- model.matrix(~0+factor(class))

colnames(design) <- c("normal","treatment")

fit <- lmFit(geo_data,design)

cont.matrix<-makeContrasts(treatment-normal,levels=design)

fit1 <- contrasts.fit(fit, cont.matrix)

fit1 <- eBayes(fit1)

allgene<-topTable(fit1,adjust='fdr',number=100000)

write.table(allgene,"allgene.xls",sep="\t",quote=F)

normaldata<-allgene[order(allgene$logFC),]

normaldata1<-rbind(Gene=colnames(normaldata),normaldata)

write.table(normaldata,"normaldata.txt",sep="\t",quote=F,col.names=F)

diffgene <- allgene[with(allgene, (abs(logFC)>=1 & P.Value < 0.05 )), ]

write.table(diffgene,"diffgene.xls",sep="\t",quote=F)

Upgene <- allgene[with(allgene, (logFC>=1 & P.Value < 0.05 )), ]

write.table(Upgene,"upgene.xls",sep="\t",quote=F)

Downgene <- allgene[with(allgene, (logFC<=(-1) & P.Value < 0.05 )), ]

write.table(Downgene,"down.xls",sep="\t",quote=F)

diffexp=geo_data[rownames(diffgene),]

diffexp1=rbind(id=colnames(diffexp),diffexp)

write.table(diffexp1,"diffexp.txt",sep="\t",quote=F,col.names=F)

inputfile_vol="vol.txt"

mydata<-read.table(inputfile_vol,header=T,row.names=1,check.names=F)

down <- mydata[mydata$logFC <= -1 & mydata$P.Value<0.05,]

up <- mydata[mydata$logFC >= 1 & mydata$P.Value<0.05,]

no <- mydata[(mydata$logFC > -1 & mydata$logFC <1),]

down<- transform(down,P.Value=-log10(down$P.Value))

up<- transform(up,P.Value=-log10(up$P.Value))

no<- transform(no,P.Value=-log10(no$P.Value))

pdf("vol1.pdf")

xm=max(abs(mydata$logFC))

ym=max(-log10(mydata$P.Value))

plot(no$logFC,no$P.Value,xlim = c(-xm,xm),ylim=c(0,ym),col="black",pch=16,cex=0.9,main = "Volcano",xlab = "logFC",ylab="-log10(P.Value)")

points(up$logFC,up$P.Value,col="red",pch=16,cex=0.9)

points(down$logFC,down$P.Value,col="blue",pch=16,cex=0.9)

abline(v=0,lwd=2)

dev.off()

pdf("vol2.pdf")

xm=max(abs(mydata$logFC))

ym=max(-log10(mydata$P.Value))

plot(no$P.Value,no$logFC,xlim = c(0,ym),ylim=c(-xm,xm),col="black",pch=16,cex=0.9,main = "Volcano",xlab = "-log10(P.Value)",ylab="logFC")

points(up$P.Value,up$logFC,col="red",pch=16,cex=0.9)

points(down$P.Value,down$logFC,col="green",pch=16,cex=0.9)

abline(h=0,lwd=1)

dev.off()

inputheatmap="diffexp.txt"

geo_data=log2(geo_data)

library(pheatmap)

data<-read.table(inputheatmap,sep="\t",header=T,row.names=1,check.names=F)

#data<-data[1:nrow(data),]

data<-data[1:40,]

pdf("heatmap.pdf")

pheatmap(data,display_numbers = F,fontsize_row=7,fontsize_col=10,cluster_cols = T,cluster_rows = T,color = colorRampPalette(c("green", "black", "red"))(50))

dev.off()

pdf("heatmap2.pdf")

annotation=read.table("group.txt",sep="\t",header=T,row.names=1)

pheatmap(data, display_numbers = F,annotation=annotation,fontsize_row=7,fontsize_col=10,color = colorRampPalette(c("green", "black", "red"))(50),

cluster_cols = T,cluster_rows = T,)

dev.off()

1. **The expression levels of differential ARGs were extracted**

library(limma)

expFile="mRNA.txt"

geneFile="gene.txt"

setwd("")

rt=read.table(expFile, header=T, sep="\t", check.names=F)

rt=as.matrix(rt)

rownames(rt)=rt[,1]

exp=rt[,2:ncol(rt)]

dimnames=list(rownames(exp),colnames(exp))

data=matrix(as.numeric(as.matrix(exp)),nrow=nrow(exp),dimnames=dimnames)

data=avereps(data)

data=data[rowMeans(data)>0,]

gene=read.table(geneFile, header=F, check.names=F, sep="\t")

sameGene=intersect(as.vector(gene[,1]), rownames(data))

geneExp=data[sameGene,]

out=rbind(ID=colnames(geneExp),geneExp)

write.table(out,file=" ",sep="\t",quote=F,col.names=F)

1. **Differential ARGs were pooled with survival data**

library(limma)

lncFile=" "

cliFile=" "

setwd("")

rt=read.table(lncFile, header=T, sep="\t", check.names=F)

rt=as.matrix(rt)

rownames(rt)=rt[,1]

exp=rt[,2:ncol(rt)]

dimnames=list(rownames(exp), colnames(exp))

data=matrix(as.numeric(as.matrix(exp)), nrow=nrow(exp), dimnames=dimnames)

data=avereps(data)

group=sapply(strsplit(colnames(data),"\\-"),"[",4)

group=sapply(strsplit(group,""),"[",1)

group=gsub("2","1",group)

data=data[,group==0]

colnames(data)=gsub("(.*?)\\-(.*?)\\-(.*?)\\-(.*?)\\-.*", "\\1\\-\\2\\-\\3", colnames(data))

data=t(data)

data=avereps(data)

cli=read.table(cliFile,header=T,sep="\t",check.names=F,row.names=1)

sameSample=intersect(row.names(data),row.names(cli))

data=data[sameSample,]

cli=cli[sameSample,]

out=cbind(cli,data)

out=cbind(id=row.names(out),out)

write.table(out,file=" ",sep="\t",row.names=F,quote=F)

1. **Prognostic correlation with ARGs**

library(survival)

coxPfilter=0.05

inputFile=" "

setwd("")

rt=read.table(inputFile, header=T, sep="\t", check.names=F, row.names=1) #

rt$futime=rt$futime/365

outTab=data.frame()

sigGenes=c("futime","fustat")

for(i in colnames(rt[,3:ncol(rt)])){

cox <- coxph(Surv(futime, fustat) ~ rt[,i], data = rt)

coxSummary = summary(cox)

coxP=coxSummary$coefficients[,"Pr(>|z|)"]

if(coxP<coxPfilter){

sigGenes=c(sigGenes,i)

outTab=rbind(outTab,

cbind(id=i,

HR=coxSummary$conf.int[,"exp(coef)"],

HR.95L=coxSummary$conf.int[,"lower .95"],

HR.95H=coxSummary$conf.int[,"upper .95"],

pvalue=coxSummary$coefficients[,"Pr(>|z|)"])

)

}

}

write.table(outTab,file=" ",sep="\t",row.names=F,quote=F)

uniSigExp=rt[,sigGenes]

uniSigExp=cbind(id=row.names(uniSigExp),uniSigExp)

write.table(uniSigExp,file="tcga.uniSigExp.txt",sep="\t",row.names=F,quote=F)

bioForest=function(coxFile=null,forestFile=null,forestCol=null){

rt <- read.table(coxFile,header=T,sep="\t",row.names=1,check.names=F)

gene <- rownames(rt)

hr <- sprintf("%.3f",rt$"HR")

hrLow <- sprintf("%.3f",rt$"HR.95L")

hrHigh <- sprintf("%.3f",rt$"HR.95H")

Hazard.ratio <- paste0(hr,"(",hrLow,"-",hrHigh,")")

pVal <- ifelse(rt$pvalue<0.001, "<0.001", sprintf("%.3f", rt$pvalue))

height=nrow(rt)/12.5+5

pdf(file=forestFile, width = 7,height = height)

n <- nrow(rt)

nRow <- n+1

ylim <- c(1,nRow)

layout(matrix(c(1,2),nc=2),width=c(3,2.5))

xlim = c(0,3)

par(mar=c(4,2.5,2,1))

plot(1,xlim=xlim,ylim=ylim,type="n",axes=F,xlab="",ylab="")

text.cex=0.8

text(0,n:1,gene,adj=0,cex=text.cex)

text(1.5-0.5*0.2,n:1,pVal,adj=1,cex=text.cex);text(1.5-0.5*0.2,n+1,'pvalue',cex=text.cex,font=2,adj=1)

text(3,n:1,Hazard.ratio,adj=1,cex=text.cex);text(3,n+1,'Hazard ratio',cex=text.cex,font=2,adj=1,)

par(mar=c(4,1,2,1),mgp=c(2,0.5,0))

xlim = c(0,max(as.numeric(hrLow),as.numeric(hrHigh)))

plot(1,xlim=xlim,ylim=ylim,type="n",axes=F,ylab="",xaxs="i",xlab="Hazard ratio")

arrows(as.numeric(hrLow),n:1,as.numeric(hrHigh),n:1,angle=90,code=3,length=0.05,col="darkblue",lwd=2.5)

abline(v=1,col="black",lty=2,lwd=2)

boxcolor = ifelse(as.numeric(hr) > 1, forestCol[1], forestCol[2])

points(as.numeric(hr), n:1, pch = 15, col = boxcolor, cex=1.6)

axis(1)

dev.off()

}

bioForest(coxFile=" ",forestFile="forest.pdf",forestCol=c("red","green"))

1. **Construction of prognostic model**

library("glmnet")

library("survival")

coxSigFile=" "

geoFile=" "

setwd("")

rt=read.table(coxSigFile, header=T, sep="\t", check.names=F, row.names=1)

geo=read.table(geoFile, header=T, sep="\t", check.names=F, row.names=1)

sameGene=intersect(colnames(rt)[3:ncol(rt)], colnames(geo)[3:ncol(geo)])

rt=rt[,c("futime","fustat",sameGene)]

rt$futime[rt$futime<=0]=0.003

x=as.matrix(rt[,c(3:ncol(rt))])

y=data.matrix(Surv(rt$futime, rt$fustat))

fit=glmnet(x, y, family="cox", maxit=1000)

pdf("lasso.lambda.pdf")

plot(fit, xvar = "lambda", label = TRUE)

dev.off()

cvfit=cv.glmnet(x, y, family="cox", maxit=1000)

pdf("lasso.cvfit.pdf")

plot(cvfit)

abline(v=log(c(cvfit$lambda.min,cvfit$lambda.1se)),lty="dashed")

dev.off()

coef=coef(fit, s=cvfit$lambda.min)

index=which(coef != 0)

actCoef=coef[index]

lassoGene=row.names(coef)[index]

geneCoef=cbind(Gene=lassoGene, Coef=actCoef)

write.table(geneCoef, file="lasso.geneCoef.txt", sep="\t", quote=F, row.names=F)

trainFinalGeneExp=rt[,lassoGene]

myFun=function(x){crossprod(as.numeric(x),actCoef)}

trainScore=apply(trainFinalGeneExp,1,myFun)

outCol=c("futime","fustat",lassoGene)

risk=as.vector(ifelse(trainScore>median(trainScore),"high","low"))

outTab=cbind(rt[,outCol],riskScore=as.vector(trainScore),risk)

write.table(cbind(id=rownames(outTab),outTab),file="trainRisk.txt",sep="\t",quote=F,row.names=F)

rt=read.table(geoFile, header=T, sep="\t", check.names=F, row.names=1)

rt$futime=rt$futime/365

testFinalGeneExp=rt[,lassoGene]

testScore=apply(testFinalGeneExp,1,myFun)

outCol=c("futime","fustat",lassoGene)

risk=as.vector(ifelse(testScore>median(trainScore),"high","low"))

outTab=cbind(rt[,outCol],riskScore=as.vector(testScore),risk)

write.table(cbind(id=rownames(outTab),outTab),file="testRisk.txt",sep="\t",quote=F,row.names=F)

1. **Survival analysis**

library(survival)

library(survminer)

setwd("")

bioSurvival=function(inputFile=null,outFile=null){

rt=read.table(inputFile, header=T, sep="\t", check.names=F)

diff=survdiff(Surv(futime, fustat) ~risk,data = rt)

pValue=1-pchisq(diff$chisq,df=1)

if(pValue<0.001){

pValue="p<0.001"

}else{

pValue=paste0("p=",sprintf("%.03f",pValue))

}

fit <- survfit(Surv(futime, fustat) ~ risk, data = rt)

surPlot=ggsurvplot(fit,

data=rt,

conf.int=T,

pval=pValue,

pval.size=6,

legend.title="Risk",

legend.labs=c("High risk", "Low risk"),

xlab="Time(years)",

break.time.by = 1,

palette=c("red", "blue"),

risk.table=TRUE,

risk.table.title="",

risk.table.height=.25)

pdf(file=outFile,onefile = FALSE,width = 6.5,height =5.5)

print(surPlot)

dev.off()

}

bioSurvival(inputFile="trainRisk.txt", outFile="trainSurv.pdf")

bioSurvival(inputFile="testRisk.txt", outFile="testSurv.pdf")

1. **ROC curve**

library(survival)

library(survminer)

library(timeROC)

setwd("")

bioROC=function(inputFile=null, rocFile=null){

rt=read.table(inputFile, header=T, sep="\t", check.names=F)

ROC_rt=timeROC(T=rt$futime, delta=rt$fustat,

marker=rt$riskScore, cause=1,

weighting='aalen',

times=c(1,3,5), ROC=TRUE)

pdf(file=rocFile,width=5,height=5)

plot(ROC_rt,time=1,col='green',title=FALSE,lwd=2)

plot(ROC_rt,time=3,col='blue',add=TRUE,title=FALSE,lwd=2)

plot(ROC_rt,time=5,col='red',add=TRUE,title=FALSE,lwd=2)

legend('bottomright',

c(paste0('AUC at 1 years: ',sprintf("%.03f",ROC_rt$AUC[1])),

paste0('AUC at 3 years: ',sprintf("%.03f",ROC_rt$AUC[2])),

paste0('AUC at 5 years: ',sprintf("%.03f",ROC_rt$AUC[3]))),

col=c("green","blue","red"),lwd=2,bty = 'n')

dev.off()

}

bioROC(inputFile="trainRisk.txt", rocFile="train.ROC.pdf")

bioROC(inputFile="testRisk.txt", rocFile="test.ROC.pdf")

1. **Risk curve**

library(pheatmap)

setwd("")

bioRiskPlot=function(inputFile=null, project=null){

rt=read.table(inputFile, header=T, sep="\t", check.names=F, row.names=1)

rt=rt[order(rt$riskScore),]

riskClass=rt[,"risk"]

lowLength=length(riskClass[riskClass=="low"])

highLength=length(riskClass[riskClass=="high"])

lowMax=max(rt$riskScore[riskClass=="low"])

line=rt[,"riskScore"]

line[line>10]=10

pdf(file=paste0(project, ".riskScore.pdf"), width=7, height=4)

plot(line, type="p", pch=20,

xlab="Patients (increasing risk socre)",

ylab="Risk score",

col=c(rep("blue",lowLength),rep("red",highLength)) )

abline(h=lowMax,v=lowLength,lty=2)

legend("topleft", c("High risk","Low Risk"),bty="n",pch=19,col=c("red","blue"),cex=1.2)

dev.off()

color=as.vector(rt$fustat)

color[color==1]="red"

color[color==0]="blue"

pdf(file=paste0(project, ".survStat.pdf"), width=7, height=4)

plot(rt$futime, pch=19,

xlab="Patients (increasing risk socre)",

ylab="Survival time (years)",

col=color)

legend("topleft", c("Dead","Alive"),bty="n",pch=19,col=c("red","blue"),cex=1.2)

abline(v=lowLength,lty=2)

dev.off()

ann_colors=list()

bioCol=c("blue", "red")

names(bioCol)=c("low", "high")

ann_colors[["Risk"]]=bioCol

rt1=rt[c(3:(ncol(rt)-2))]

rt1=t(rt1)

annotation=data.frame(Risk=rt[,ncol(rt)])

rownames(annotation)=rownames(rt)

pdf(file=paste0(project, ".heatmap.pdf"), width=7, height=4)

pheatmap(rt1,

annotation=annotation,

annotation_colors = ann_colors,

cluster_cols = FALSE,

cluster_rows = FALSE,

show_colnames = F,

scale="row",

color = colorRampPalette(c(rep("blue",3.5), "white", rep("red",3.5)))(50),

fontsize_col=3,

fontsize=7,

fontsize_row=8)

dev.off()

}

bioRiskPlot(inputFile="risk.train.txt", project="train")

bioRiskPlot(inputFile="risk.test.txt", project="test")

bioRiskPlot(inputFile="risk.all.txt", project="all")

1. **Independent prognostic analysis**

library(survival)

setwd("")

bioForest=function(coxFile=null, forestFile=null, forestCol=null){

rt <- read.table(coxFile, header=T, sep="\t", check.names=F, row.names=1)

gene <- rownames(rt)

hr <- sprintf("%.3f",rt$"HR")

hrLow <- sprintf("%.3f",rt$"HR.95L")

hrHigh <- sprintf("%.3f",rt$"HR.95H")

Hazard.ratio <- paste0(hr,"(",hrLow,"-",hrHigh,")")

pVal <- ifelse(rt$pvalue<0.001, "<0.001", sprintf("%.3f", rt$pvalue))

pdf(file=forestFile, width=6.6, height=4.5)

n <- nrow(rt)

nRow <- n+1

ylim <- c(1,nRow)

layout(matrix(c(1,2),nc=2),width=c(3,2.5))

xlim = c(0,3)

par(mar=c(4,2.5,2,1))

plot(1,xlim=xlim,ylim=ylim,type="n",axes=F,xlab="",ylab="")

text.cex=0.8

text(0,n:1,gene,adj=0,cex=text.cex)

text(1.5-0.5*0.2,n:1,pVal,adj=1,cex=text.cex);text(1.5-0.5*0.2,n+1,'pvalue',cex=text.cex,font=2,adj=1)

text(3.1,n:1,Hazard.ratio,adj=1,cex=text.cex);text(3.1,n+1,'Hazard ratio',cex=text.cex,font=2,adj=1)

par(mar=c(4,1,2,1),mgp=c(2,0.5,0))

xlim = c(0,max(as.numeric(hrLow),as.numeric(hrHigh)))

plot(1,xlim=xlim,ylim=ylim,type="n",axes=F,ylab="",xaxs="i",xlab="Hazard ratio")

arrows(as.numeric(hrLow),n:1,as.numeric(hrHigh),n:1,angle=90,code=3,length=0.05,col="darkblue",lwd=2.5)

abline(v=1,col="black",lty=2,lwd=2)

boxcolor = ifelse(as.numeric(hr) > 1, forestCol, forestCol)

points(as.numeric(hr), n:1, pch = 15, col = boxcolor, cex=1.5)

axis(1)

dev.off()

}

indep=function(riskFile=null, cliFile=null, project=null){

risk=read.table(riskFile, header=T, sep="\t", check.names=F, row.names=1)

cli=read.table(cliFile, header=T, sep="\t", check.names=F, row.names=1)

sameSample=intersect(row.names(cli),row.names(risk))

risk=risk[sameSample,]

cli=cli[sameSample,]

rt=cbind(futime=risk[,1], fustat=risk[,2], cli, riskScore=risk[,(ncol(risk)-1)])

uniCoxFile=paste0(project,".uniCox.txt")

uniCoxPdf=paste0(project,".uniCox.pdf")

uniTab=data.frame()

for(i in colnames(rt[,3:ncol(rt)])){

cox <- coxph(Surv(futime, fustat) ~ rt[,i], data = rt)

coxSummary = summary(cox)

uniTab=rbind(uniTab,

cbind(id=i,

HR=coxSummary$conf.int[,"exp(coef)"],

HR.95L=coxSummary$conf.int[,"lower .95"],

HR.95H=coxSummary$conf.int[,"upper .95"],

pvalue=coxSummary$coefficients[,"Pr(>|z|)"])

)

}

write.table(uniTab,file=uniCoxFile,sep="\t",row.names=F,quote=F)

bioForest(coxFile=uniCoxFile, forestFile=uniCoxPdf, forestCol="green")

multiCoxFile=paste0(project,".multiCox.txt")

multiCoxPdf=paste0(project,".multiCox.pdf")

uniTab=uniTab[as.numeric(uniTab[,"pvalue"])<1,]

rt1=rt[,c("futime","fustat",as.vector(uniTab[,"id"]))]

multiCox=coxph(Surv(futime, fustat) ~ ., data = rt1)

multiCoxSum=summary(multiCox)

multiTab=data.frame()

multiTab=cbind(

HR=multiCoxSum$conf.int[,"exp(coef)"],

HR.95L=multiCoxSum$conf.int[,"lower .95"],

HR.95H=multiCoxSum$conf.int[,"upper .95"],

pvalue=multiCoxSum$coefficients[,"Pr(>|z|)"])

multiTab=cbind(id=row.names(multiTab),multiTab)

write.table(multiTab, file=multiCoxFile, sep="\t", row.names=F, quote=F)

bioForest(coxFile=multiCoxFile, forestFile=multiCoxPdf, forestCol="red")

}

indep(riskFile=" ", cliFile=" ", project="all")

1. **Nomogram**

library(survival)

library(regplot)

library(rms)

riskFile=" "

cliFile=" "

setwd("")

risk=read.table(riskFile, header=T, sep="\t", check.names=F, row.names=1)

cli=read.table(cliFile, header=T, sep="\t", check.names=F, row.names=1)

cli=cli[apply(cli,1,function(x)any(is.na(match('unknow',x)))),,drop=F]

cli$Age=as.numeric(cli$Age)

samSample=intersect(row.names(risk), row.names(cli))

risk1=risk[samSample,,drop=F]

cli=cli[samSample,,drop=F]

rt=cbind(risk1[,c("futime", "fustat", "risk")], cli)

res.cox=coxph(Surv(futime, fustat) ~ . , data = rt)

nom1=regplot(res.cox,

plots = c("density", "boxes"),

clickable=F,

title="",

points=TRUE,

droplines=TRUE,

observation=rt[2,],

rank="sd",

failtime = c(1,3,5),

prfail = F)

nomoRisk=predict(res.cox, data=rt, type="risk")

rt=cbind(risk1, Nomogram=nomoRisk)

outTab=rbind(ID=colnames(rt), rt)

write.table(outTab, file="nomoRisk.txt", sep="\t", col.names=F, quote=F)

pdf(file="calibration.pdf", width=5, height=5)

f <- cph(Surv(futime, fustat) ~ Nomogram, x=T, y=T, surv=T, data=rt, time.inc=1)

cal <- calibrate(f, cmethod="KM", method="boot", u=1, m=(nrow(rt)/3), B=1000)

plot(cal, xlim=c(0,1), ylim=c(0,1),

xlab="Nomogram-predicted OS (%)", ylab="Observed OS (%)", lwd=1.5, col="green", sub=F)

f <- cph(Surv(futime, fustat) ~ Nomogram, x=T, y=T, surv=T, data=rt, time.inc=3)

cal <- calibrate(f, cmethod="KM", method="boot", u=3, m=(nrow(rt)/3), B=1000)

plot(cal, xlim=c(0,1), ylim=c(0,1), xlab="", ylab="", lwd=1.5, col="blue", sub=F, add=T)

f <- cph(Surv(futime, fustat) ~ Nomogram, x=T, y=T, surv=T, data=rt, time.inc=5)

cal <- calibrate(f, cmethod="KM", method="boot", u=5, m=(nrow(rt)/3), B=1000)

plot(cal, xlim=c(0,1), ylim=c(0,1), xlab="", ylab="", lwd=1.5, col="red", sub=F, add=T)

legend('bottomright', c('1-year', '3-year', '5-year'),

col=c("green","blue","red"), lwd=1.5, bty = 'n')

dev.off()

1. **Tumor microenvironment**

library(limma)

library(estimate)

inputFile=" "

setwd("D ")

rt=read.table(inputFile, header=T, sep="\t", check.names=F)

rt=as.matrix(rt)

rownames(rt)=rt[,1]

exp=rt[,2:ncol(rt)]

dimnames=list(rownames(exp), colnames(exp))

data=matrix(as.numeric(as.matrix(exp)), nrow=nrow(exp), dimnames=dimnames)

data=avereps(data)

group=sapply(strsplit(colnames(data),"\\-"), "[", 4)

group=sapply(strsplit(group,""), "[", 1)

group=gsub("2", "1", group)

data=data[,group==0]

out=data[rowMeans(data)>0,]

out=rbind(ID=colnames(out), out)

write.table(out,file="uniq.symbol.txt",sep="\t",quote=F,col.names=F)

filterCommonGenes(input.f="uniq.symbol.txt",

output.f="commonGenes.gct",

id="GeneSymbol")

estimateScore(input.ds = "commonGenes.gct",

output.ds="estimateScore.gct",

platform="illumina")

scores=read.table("estimateScore.gct", skip=2, header=T)

rownames(scores)=scores[,1]

scores=t(scores[,3:ncol(scores)])

rownames(scores)=gsub("\\.", "\\-", rownames(scores))

out=rbind(ID=colnames(scores), scores)

write.table(out,file="scores.txt", sep="\t", quote=F, col.names=F)

1. **Differential analysis of tumor microenvironment**

library(limma)

library(ggpubr)

scoreFile=" "

riskFile=" "

setwd("")

rt=read.table(scoreFile, header=T, sep="\t", check.names=F, row.names=1)

data=as.matrix(rt)

rownames(data)=gsub("(.*?)\\-(.*?)\\-(.*?)\\-(.*?)\\-.*", "\\1\\-\\2\\-\\3", rownames(data))

data=avereps(data)

risk=read.table(riskFile, header=T, sep="\t", check.names=F, row.names=1)

sameSample=intersect(row.names(data), row.names(risk))

data=data[sameSample,,drop=F]

risk=risk[sameSample,"risk",drop=F]

rt=cbind(data, risk)

rt$risk=factor(rt$risk, levels=c("low", "high"))

group=levels(factor(rt$risk))

rt$risk=factor(rt$risk, levels=group)

comp=combn(group,2)

my_comparisons=list()

for(i in 1:ncol(comp)){my_comparisons[[i]]<-comp[,i]}

for(i in colnames(rt)[1:3]){

gg1=ggviolin(rt, x="risk", y=i, fill = "risk",

xlab="", ylab=i,

legend.title="Risk",

palette=c("#0066FF", "#FF0000"),

add = "boxplot", add.params = list(fill="white"))+

stat_compare_means(comparisons = my_comparisons)

#stat_compare_means(comparisons = my_comparisons,symnum.args=list(cutpoints = c(0, 0.001, 0.01, 0.05, 1), symbols = c("***", "**", "*", "ns")),label = "p.signif")

pdf(file=paste0(i, ".pdf"), width=6, height=5)

print(gg1)

dev.off()

}

1. **ssGSEA**

library(GSVA)

library(limma)

library(GSEABase)

setwd("")

immuneScore=function(expFile=null, gmtFile=null, project=null){

rt=read.table(expFile, header=T, sep="\t", check.names=F)

rt=as.matrix(rt)

rownames(rt)=rt[,1]

exp=rt[,2:ncol(rt)]

dimnames=list(rownames(exp),colnames(exp))

mat=matrix(as.numeric(as.matrix(exp)),nrow=nrow(exp),dimnames=dimnames)

mat=avereps(mat)

mat=mat[rowMeans(mat)>0,]

geneSet=getGmt(gmtFile, geneIdType=SymbolIdentifier())

ssgseaScore=gsva(mat, geneSet, method='ssgsea', kcdf='Gaussian', abs.ranking=TRUE)

normalize=function(x){

return((x-min(x))/(max(x)-min(x)))}

ssgseaOut=normalize(ssgseaScore)

ssgseaOut=rbind(id=colnames(ssgseaOut),ssgseaOut)

write.table(ssgseaOut, file=paste0(project, ".score.txt"), sep="\t", quote=F, col.names=F)

}

immuneScore(expFile=" ", gmtFile="immune.gmt", project=" ")

1. **Differential analysis of ssGSEA**

library(limma)

library(reshape2)

library(ggpubr)

setwd("")

scoreCor=function(riskFile=null, scoreFile=null, project=null){

data=read.table(scoreFile, header=T, sep="\t", check.names=F, row.names=1)

data=t(data)

risk=read.table(riskFile, header=T, sep="\t", check.names=F, row.names=1)

sameSample=intersect(row.names(data),row.names(risk))

data=data[sameSample,,drop=F]

risk=risk[sameSample,,drop=F]

rt=cbind(data,risk[,c("riskScore","risk")])

rt=rt[,-(ncol(rt)-1)]

immCell=c("aDCs","B_cells","CD8+_T_cells","DCs","iDCs","Macrophages",

"Mast_cells","Neutrophils","NK_cells","pDCs","T_helper_cells",

"Tfh","Th1_cells","Th2_cells","TIL","Treg")

rt1=rt[,c(immCell,"risk")]

data=melt(rt1,id.vars=c("risk"))

colnames(data)=c("Risk","Type","Score")

data$Risk=factor(data$Risk, levels=c("low","high"))

p=ggboxplot(data, x="Type", y="Score", color = "Risk",

xlab="",ylab="Score",add = "none",palette = c("blue","red") )

p=p+rotate_x_text(50)

p=p+stat_compare_means(aes(group=Risk),symnum.args=list(cutpoints = c(0, 0.001, 0.01, 0.05, 1), symbols = c("***", "**", "*", "")),label = "p.signif")

pdf(file=paste0(project,".immCell.pdf"), width=7, height=6)

print(p)

dev.off()

immFunction=c("APC_co_inhibition","APC_co_stimulation","CCR",

"Check-point","Cytolytic_activity","HLA","Inflammation-promoting",

"MHC_class_I","Parainflammation","T_cell_co-inhibition",

"T_cell_co-stimulation","Type_I_IFN_Reponse","Type_II_IFN_Reponse")

rt1=rt[,c(immFunction,"risk")]

data=melt(rt1,id.vars=c("risk"))

colnames(data)=c("Risk","Type","Score")

data$Risk=factor(data$Risk, levels=c("low","high"))

p=ggboxplot(data, x="Type", y="Score", color = "Risk",

xlab="",ylab="Score",add = "none",palette = c("blue","red") )

p=p+rotate_x_text(50)

p=p+stat_compare_means(aes(group=Risk),symnum.args=list(cutpoints = c(0, 0.001, 0.01, 0.05, 1), symbols = c("***", "**", "*", "")),label = "p.signif")

pdf(file=paste0(project,".immFunction.pdf"), width=7, height=6)

print(p)

dev.off()

}

scoreCor(riskFile=" ", scoreFile=" ", project=" ")

1. **Infiltration of immune cells**

library(e1071)

library(parallel)

library(preprocessCore)

X <- read.table(sig_matrix,header=T,sep="\t",row.names=1,check.names=F)

Y <- read.table(mixture_file, header=T, sep="\t", row.names=1,check.names=F)

X <- data.matrix(X)

Y <- data.matrix(Y)

#order

X <- X[order(rownames(X)),]

Y <- Y[order(rownames(Y)),]

P <- perm #number of permutations

#anti-log if max < 50 in mixture file

if(max(Y) < 50) {Y <- 2^Y}

#quantile normalization of mixture file

if(QN == TRUE){

tmpc <- colnames(Y)

tmpr <- rownames(Y)

Y <- normalize.quantiles(Y)

colnames(Y) <- tmpc

rownames(Y) <- tmpr

}

#intersect genes

Xgns <- row.names(X)

Ygns <- row.names(Y)

YintX <- Ygns %in% Xgns

Y <- Y[YintX,]

XintY <- Xgns %in% row.names(Y)

X <- X[XintY,]

#standardize sig matrix

X <- (X - mean(X)) / sd(as.vector(X))

#empirical null distribution of correlation coefficients

if(P > 0) {nulldist <- sort(doPerm(P, X, Y)$dist)}

#print(nulldist)

header <- c('Mixture',colnames(X),"P-value","Correlation","RMSE")

#print(header)

output <- matrix()

itor <- 1

mixtures <- dim(Y)[2]

pval <- 9999

#iterate through mixtures

while(itor <= mixtures){

y <- Y[,itor]

#standardize mixture

y <- (y - mean(y)) / sd(y)

#run SVR core algorithm

result <- CoreAlg(X, y)

#get results

w <- result$w

mix_r <- result$mix_r

mix_rmse <- result$mix_rmse

#calculate p-value

if(P > 0) {pval <- 1 - (which.min(abs(nulldist - mix_r)) / length(nulldist))}

#print output

out <- c(colnames(Y)[itor],w,pval,mix_r,mix_rmse)

if(itor == 1) {output <- out}

else {output <- rbind(output, out)}

itor <- itor + 1

}

write.table(rbind(header,output), file="CIBERSORT-Results.txt", sep="\t", row.names=F, col.names=F, quote=F)

1. **Immune cell correlation analysis**

library(limma)

library(reshape2)

library(ggpubr)

library(ggExtra)

gene=" "

expFile="normalize.txt"

immFile="CIBERSORT-Results.txt"

setwd("")

rt=read.table(expFile, header=T, sep="\t", check.names=F)

rt=as.matrix(rt)

rownames(rt)=rt[,1]

exp=rt[,2:ncol(rt)]

dimnames=list(rownames(exp),colnames(exp))

data=matrix(as.numeric(as.matrix(exp)),nrow=nrow(exp),dimnames=dimnames)

data=avereps(data)

data=t(data[gene,,drop=F])

data=as.data.frame(data)

immune=read.table(immFile, header=T, sep="\t", check.names=F, row.names=1)

sameSample=intersect(row.names(immune), row.names(data))

rt=cbind(immune[sameSample,,drop=F], data[sameSample,,drop=F])

outTab=data.frame()

for(i in colnames(rt)[1:(ncol(rt)-1)]){

x=as.numeric(rt[,gene])

y=as.numeric(rt[,i])

if(sd(y)==0){y[1]=0.00001}

cor=cor.test(x, y, method="spearma")

outVector=cbind(Gene=gene, Cell=i, cor=cor$estimate, pvalue=cor$p.value)

outTab=rbind(outTab,outVector)

if(cor$p.value<0.05){

outFile=paste0("cor.", i, ".pdf")

df1=as.data.frame(cbind(x,y))

p1=ggplot(df1, aes(x, y)) +

xlab(paste0(gene, " expression")) + ylab(i)+

geom_point() + geom_smooth(method="lm",formula = y ~ x) + theme_bw()+

stat_cor(method = 'spearman', aes(x =x, y =y))

p2=ggMarginal(p1, type="density", xparams=list(fill = "orange"), yparams=list(fill = "blue"))

pdf(file=outFile, width=5.2, height=5)

print(p2)

dev.off()

}

}

write.table(outTab,file="cor.result.txt",sep="\t",row.names=F,quote=F)

inputFile="cor.result.txt"

setwd("")

data = read.table(inputFile, header=T, sep="\t", check.names=F)

p.col = c('gold','pink','orange','LimeGreen','darkgreen')

fcolor = function(x,p.col){

color = ifelse(x>0.8,p.col[1],ifelse(x>0.6,p.col[2],ifelse(x>0.4,p.col[3],

ifelse(x>0.2,p.col[4], p.col[5])

)))

return(color)

}

p.cex = seq(2.5, 5.5, length=5)

fcex = function(x){

x=abs(x)

cex = ifelse(x<0.1,p.cex[1],ifelse(x<0.2,p.cex[2],ifelse(x<0.3,p.cex[3],

ifelse(x<0.4,p.cex[4],p.cex[5]))))

return(cex)

}

points.color = fcolor(x=data$pvalue,p.col=p.col)

data$points.color = points.color

points.cex = fcex(x=data$cor)

data$points.cex = points.cex

data=data[order(data$cor),]

xlim = ceiling(max(abs(data$cor))*10)/10

pdf(file="Lollipop.pdf", width=9, height=7)

layout(mat=matrix(c(1,1,1,1,1,0,2,0,3,0),nc=2),width=c(8,2.2),heights=c(1,2,1,2,1))

par(bg="white",las=1,mar=c(5,18,2,4),cex.axis=1.5,cex.lab=2)

plot(1,type="n",xlim=c(-xlim,xlim),ylim=c(0.5,nrow(data)+0.5),xlab="Correlation Coefficient",ylab="",yaxt="n",yaxs="i",axes=F)

rect(par('usr')[1],par('usr')[3],par('usr')[2],par('usr')[4],col="#F5F5F5",border="#F5F5F5")

grid(ny=nrow(data),col="white",lty=1,lwd=2)

segments(x0=data$cor,y0=1:nrow(data),x1=0,y1=1:nrow(data),lwd=4)

points(x=data$cor,y = 1:nrow(data),col = data$points.color,pch=16,cex=data$points.cex)

text(par('usr')[1],1:nrow(data),data$Cell,adj=1,xpd=T,cex=1.5)

pvalue.text=ifelse(data$pvalue<0.001,'<0.001',sprintf("%.03f",data$pvalue))

redcutoff_cor=0

redcutoff_pvalue=0.05

text(par('usr')[2],1:nrow(data),pvalue.text,adj=0,xpd=T,col=ifelse(abs(data$cor)>redcutoff_cor & data$pvalue<redcutoff_pvalue,"red","black"),cex=1.5)

axis(1,tick=F)

par(mar=c(0,4,3,4))

plot(1,type="n",axes=F,xlab="",ylab="")

legend("left",legend=c(0.1,0.2,0.3,0.4,0.5),col="black",pt.cex=p.cex,pch=16,bty="n",cex=2,title="abs(cor)")

par(mar=c(0,6,4,6),cex.axis=1.5,cex.main=2)

barplot(rep(1,5),horiz=T,space=0,border=NA,col=p.col,xaxt="n",yaxt="n",xlab="",ylab="",main="pvalue")

axis(4,at=0:5,c(1,0.8,0.6,0.4,0.2,0),tick=F)

dev.off()

1. **Correlation analysis between risk and clinical traits**

library(limma)

library(ggpubr)

cliFile=" "

riskFile=" "

scoreFile=" "

setwd("")

Cluster=read.table(ClusterFile, header=F, sep="\t", check.names=F, row.names=1)

colnames(Cluster)=c("Cluster")

cli=read.table(cliFile, header=T, sep="\t", check.names=F, row.names=1)

risk=read.table(riskFile, header=T, sep="\t", check.names=F, row.names=1)

score=read.table(scoreFile, header=T, sep="\t", check.names=F, row.names=1)

row.names(score)=gsub("(.*?)\\-(.*?)\\-(.*?)\\-(.*?)\\-.*", "\\1\\-\\2\\-\\3", row.names(score))

score=avereps(score)

samSample=intersect(row.names(Cluster), row.names(cli))

Cluster=Cluster[samSample,"Cluster",drop=F]

cli=cli[samSample,,drop=F]

risk=risk[samSample,,drop=F]

score=score[samSample,,drop=F]

score[,"ImmuneScore"]=ifelse(score[,"ImmuneScore"]>median(score[,"ImmuneScore"]), "High", "Low")

data=cbind(risk, Cluster, score[,"ImmuneScore",drop=F], cli)

rt=data[order(data$riskScore),,drop=F]

rt=rt[,((ncol(risk)-1):ncol(rt))]

rt=rt[,-2]

rt$Cluster=paste0("Cluster", rt$Cluster)

for(clinical in colnames(rt[,2:ncol(rt)])){

data=rt[c("riskScore", clinical)]

colnames(data)=c("riskScore", "clinical")

data=data[(data[,"clinical"]!="unknow"),]

group=levels(factor(data$clinical))

data$clinical=factor(data$clinical, levels=group)

comp=combn(group,2)

my_comparisons=list()

for(i in 1:ncol(comp)){my_comparisons[[i]]<-comp[,i]}

boxplot=ggboxplot(data, x="clinical", y="riskScore", color="clinical",

xlab=clinical,

ylab="Risk score",

legend.title=clinical,

add = "jitter")+

stat_compare_means(comparisons = my_comparisons)

pdf(file=paste0(clinical, ".pdf"), width=5.5, height=5)

print(boxplot)

dev.off()

}

1. **Immune checkpoint analysis**

library(limma)

library(reshape2)

library(ggplot2)

library(ggpubr)

expFile=" "

riskFile=" "

geneFile=" "

setwd("")

rt=read.table(expFile, header=T, sep="\t", check.names=F)

rt=as.matrix(rt)

rownames(rt)=rt[,1]

exp=rt[,2:ncol(rt)]

dimnames=list(rownames(exp),colnames(exp))

data=matrix(as.numeric(as.matrix(exp)),nrow=nrow(exp),dimnames=dimnames)

data=avereps(data)

gene=read.table(geneFile, header=F, sep="\t", check.names=F)

sameGene=intersect(row.names(data),as.vector(gene[,1]))

data=t(data[sameGene,])

data=log2(data+1)

group=sapply(strsplit(row.names(data),"\\-"),"[",4)

group=sapply(strsplit(group,""),"[",1)

group=gsub("2","1",group)

data=data[group==0,]

row.names(data)=gsub("(.*?)\\-(.*?)\\-(.*?)\\-(.*?)\\-.*","\\1\\-\\2\\-\\3",row.names(data))

data=avereps(data)

risk=read.table(riskFile, sep="\t", header=T, check.names=F, row.names=1)

sameSample=intersect(row.names(data),row.names(risk))

rt1=cbind(data[sameSample,],risk[sameSample,])

rt1=rt1[,c(sameGene,"risk")]

sigGene=c()

for(i in colnames(rt1)[1:(ncol(rt1)-1)]){

if(sd(rt1[,i])<0.001){next}

wilcoxTest=wilcox.test(rt1[,i] ~ rt1[,"risk"])

pvalue=wilcoxTest$p.value

if(wilcoxTest$p.value<0.05){

sigGene=c(sigGene, i)

}

}

sigGene=c(sigGene, "risk")

rt1=rt1[,sigGene]

rt1=melt(rt1,id.vars=c("risk"))

colnames(rt1)=c("risk","Gene","Expression")

group=levels(factor(rt1$risk))

rt1$risk=factor(rt1$risk, levels=c("low","high"))

comp=combn(group,2)

my_comparisons=list()

for(j in 1:ncol(comp)){my_comparisons[[j]]<-comp[,j]}

boxplot=ggboxplot(rt1, x="Gene", y="Expression", fill="risk",

xlab="",

ylab="Gene expression",

legend.title="Risk",

width=0.8,

palette = c("#0066FF", "#FF0000") )+

rotate_x_text(50)+

stat_compare_means(aes(group=risk),

method="wilcox.test",

symnum.args=list(cutpoints=c(0, 0.001, 0.01, 0.05, 1), symbols=c("***", "**", "*", "ns")), label="p.signif")

pdf(file="checkpoint.diff.pdf", width=8, height=5)

print(boxplot)

dev.off()

1. **Drug sensitivity analysis**

library(limma)

library(ggpubr)

library(pRRophetic)

library(ggplot2)

set.seed(12345)

pFilter=0.001

expFile=" "

riskFile=" "

setwd("")

allDrugs=c("A.443654", "A.770041", "ABT.263", "ABT.888", "AG.014699", "AICAR", "AKT.inhibitor.VIII", "AMG.706", "AP.24534", "AS601245", "ATRA", "AUY922", "Axitinib", "AZ628", "AZD.0530", "AZD.2281", "AZD6244", "AZD6482", "AZD7762", "AZD8055", "BAY.61.3606", "Bexarotene", "BI.2536", "BIBW2992", "Bicalutamide", "BI.D1870", "BIRB.0796", "Bleomycin", "BMS.509744", "BMS.536924", "BMS.708163", "BMS.754807", "Bortezomib", "Bosutinib", "Bryostatin.1", "BX.795", "Camptothecin", "CCT007093", "CCT018159", "CEP.701", "CGP.082996", "CGP.60474", "CHIR.99021", "CI.1040", "Cisplatin", "CMK", "Cyclopamine", "Cytarabine", "Dasatinib", "DMOG", "Docetaxel", "Doxorubicin", "EHT.1864", "Elesclomol", "Embelin", "Epothilone.B", "Erlotinib", "Etoposide", "FH535", "FTI.277", "GDC.0449", "GDC0941", "Gefitinib", "Gemcitabine", "GNF.2", "GSK269962A", "GSK.650394", "GW.441756", "GW843682X", "Imatinib", "IPA.3", "JNJ.26854165", "JNK.9L", "JNK.Inhibitor.VIII", "JW.7.52.1", "KIN001.135", "KU.55933", "Lapatinib", "Lenalidomide", "LFM.A13", "Metformin", "Methotrexate", "MG.132", "Midostaurin", "Mitomycin.C", "MK.2206", "MS.275", "Nilotinib", "NSC.87877", "NU.7441", "Nutlin.3a", "NVP.BEZ235", "NVP.TAE684", "Obatoclax.Mesylate", "OSI.906", "PAC.1", "Paclitaxel", "Parthenolide", "Pazopanib", "PD.0325901", "PD.0332991", "PD.173074", "PF.02341066", "PF.4708671", "PF.562271", "PHA.665752", "PLX4720", "Pyrimethamine", "QS11", "Rapamycin", "RDEA119", "RO.3306", "Roscovitine", "Salubrinal", "SB.216763", "SB590885", "Shikonin", "SL.0101.1", "Sorafenib", "S.Trityl.L.cysteine", "Sunitinib", "Temsirolimus", "Thapsigargin", "Tipifarnib", "TW.37", "Vinblastine", "Vinorelbine", "Vorinostat", "VX.680", "VX.702", "WH.4.023", "WO2009093972", "WZ.1.84", "X17.AAG", "X681640", "XMD8.85", "Z.LLNle.CHO", "ZM.447439")

rt = read.table(expFile, header=T, sep="\t", check.names=F)

rt=as.matrix(rt)

rownames(rt)=rt[,1]

exp=rt[,2:ncol(rt)]

dimnames=list(rownames(exp),colnames(exp))

data=matrix(as.numeric(as.matrix(exp)),nrow=nrow(exp),dimnames=dimnames)

data=avereps(data)

data=data[rowMeans(data)>0.5,]

group=sapply(strsplit(colnames(data),"\\-"), "[", 4)

group=sapply(strsplit(group,""), "[", 1)

group=gsub("2","1",group)

data=data[,group==0]

data=t(data)

rownames(data)=gsub("(.*?)\\-(.*?)\\-(.*?)\\-(.*)", "\\1\\-\\2\\-\\3", rownames(data))

data=avereps(data)

data=t(data)

riskRT=read.table(riskFile, header=T, sep="\t", check.names=F, row.names=1)

for(drug in allDrugs){

senstivity=pRRopheticPredict(data, drug, selection=1)

senstivity=senstivity[senstivity!="NaN"]

#senstivity[senstivity>quantile(senstivity,0.99)]=quantile(senstivity,0.99)

sameSample=intersect(row.names(riskRT), names(senstivity))

risk=riskRT[sameSample, "risk",drop=F]

senstivity=senstivity[sameSample]

rt=cbind(risk, senstivity)

rt$risk=factor(rt$risk, levels=c("low", "high"))

type=levels(factor(rt[,"risk"]))

comp=combn(type, 2)

my_comparisons=list()

for(i in 1:ncol(comp)){my_comparisons[[i]]<-comp[,i]}

test=wilcox.test(senstivity~risk, data=rt)

if(test$p.value<pFilter){

boxplot=ggboxplot(rt, x="risk", y="senstivity", fill="risk",

xlab="Risk",

ylab=paste0(drug, " senstivity (IC50)"),

legend.title="Risk",

palette=c("#0066FF","#FF0000")

)+

stat_compare_means(comparisons=my_comparisons)

pdf(file=paste0("durgSenstivity.", drug, ".pdf"), width=5, height=4.5)

print(boxplot)

dev.off()

}

}
